# Adhesion G protein–coupled receptor Gpr126/Adgrg6 is essential for placental development

**DOI:** 10.1126/sciadv.abj5445

**Published:** 2021-11-12

**Authors:** Rebeca Torregrosa-Carrión, Rebeca Piñeiro-Sabarís, Marcos Siguero-Álvarez, Joaquím Grego-Bessa, Luis Luna-Zurita, Vitor Samuel Fernandes, Donal MacGrogan, Didier Y. R. Stainier, José Luis de la Pompa

**Affiliations:** 1 Intercellular Signalling in Cardiovascular Development and Disease Laboratory, Centro Nacional de Investigaciones Cardiovasculares (CNIC), Melchor Fernández Almagro 3, 28029 Madrid, Spain.; 2 Ciber de Enfermedades Cardiovasculares, 28029 Madrid, Spain.; 3 Department of Developmental Genetics, Max Planck Institute for Heart and Lung Research, 61231 Bad Nauheim, Germany.

## Abstract

Mutations in the G protein–coupled receptor *GPR126/ADGRG6* cause human diseases, including defective peripheral nervous system (PNS) myelination. To study GPR126 function, we generated new genetic mice and zebrafish models. Murine *Gpr126* is expressed in developing heart endocardium, and global *Gpr126* inactivation is embryonically lethal, with mutants having thin-walled ventricles but unaffected heart patterning or maturation. Endocardial-specific *Gpr126* deletion does not affect heart development or function, and transgenic endocardial *GPR126* expression fails to rescue lethality in *Gpr126*-null mice. Zebrafish *gpr126* mutants display unaffected heart development. *Gpr126* is also expressed in placental trophoblast giant cells. *Gpr126*-null mice with a heterozygous placenta survive but exhibit GPR126-defective PNS phenotype. In contrast, *Gpr126*-null embryos with homozygous mutant placenta die but are rescued by placental *GPR126* expression. *Gpr126*-deficient placentas display down-regulation of preeclampsia markers *Mmp9*, *Cts7*, and *Cts8*. We propose that the placenta-heart axis accounts for heart abnormalities secondary to placental defects in *Gpr126* mutants.

## Teaser

Mouse and zebrafish genetic experiments demonstrate GPR126 requirement for placentation and dispensability for heart development.

## INTRODUCTION

Adhesion G protein–coupled receptors (aGPCRs) are a large family of cell surface receptors that participate in signaling events through their large extracellular domain (ECD), which contains several adhesion-like domains that mediate cell-cell and cell-matrix interactions. aGPCRs also contain a characteristic seven–transmembrane helix (7TM) domain involved in canonical signaling and a short intracellular domain (*1*, *2*). The membrane-proximal portion of the ECD, adjacent to the 7TM, is a conserved GPCR autoproteolysis inducing (GAIN) domain. During receptor processing in the endoplasmic reticulum, autoproteolysis in the GAIN domain cleaves aGPCRs into N- and C-terminal fragments (NTF and CTF) that remain noncovalently associated at the cell surface (*3*). The NTF includes most of the ECD, whereas the CTF is formed by the remaining ECR, the 7TM domain, and the intracellular region (*1*). Key amino acids in the CTF portion of the GAIN domain (the *Stachel* sequence) can function as a tethered agonist for CTF activation (*2*). However, upon ligand interaction or mechanical removal of the NTF, the *Stachel* sequence is exposed and able to bind the 7TM, thus activating the G protein–signaling cascade (cis signaling) (*2*). Another mode of GPR126 activation is via the action of NTF independently of the CTF (trans activation) (*4*, *5*). The 7TM is defined by its ability to bind heterotrimeric G proteins, leading to increased production of the second messenger molecule cyclic adenosine monophosphate (cAMP), which initiates a signaling cascade via activation of cAMP-dependent protein kinases.

*Gpr126* is encoded by 25 exons in mice and zebrafish and exists in several alternatively spliced forms, which differ in the presence of exon 6 and exon 25 (*6*). The *Gpr126* locus is highly conserved in vertebrates, and the synteny of the genomic region surrounding these genes is conserved in fish, birds, reptiles, and mammals, indicating that the predicted genes are true orthologs (*7*). Gpr126 functions in multiple essential processes, including peripheral nervous system (PNS) myelination (*8*, *9*), bone formation (*10*), and inner ear development (*11*). In addition, human *GPR126* mutations are linked to several diseases, including adolescent idiopathic scoliosis (*12*) and arthrogryposis multiplex congenita (AMC) (*13*). We identified *Gpr126* as a Notch-regulated gene during cardiac chamber development in mice and suggested that endocardial Gpr126 might non–cell autonomously influence the proliferation and differentiation of adjacent cardiomyocytes within the developing ventricles (*14*). Independent proposals for a role of Gpr126 in heart development in mice (*5*, *7*) are supported by evidence from morpholino studies in zebrafish (*5*); however, the role of Gpr126 in cardiac development has not been definitely established.

In this study, we show that Gpr126 is not required for heart development and describe an essential role for Gpr126 in placentation. We show that global *Gpr126* inactivation in mice leads to embryonic death but that endocardium-specific *Gpr126* deletion does not affect cardiac development or function. In addition, *GPR126* expression in the endocardium does not rescue lethality in *Gpr126-*deficient mice, indicating that embryonic demise is not due to defective cardiac development. Accordingly, *gpr126*-deficient zebrafish mutants display wild type (WT)–like heart development. *Gpr126* is also expressed in trophoblast giant cells (TGCs) in the placenta. *Gpr126* homozygous inactivation in the embryo proper allows survival if the placenta is *Gpr126* heterozygous, but *Gpr126* inactivation in the embryo and in the placenta leads to embryonic death. *Gpr126* mutant placentas display reduced expression of proteases involved in trophoblast invasion and maternal uterine vasculature remodeling. Defective remodeling leads to an impaired fetal blood supply, intrauterine growth restriction, preeclampsia, and early pregnancy loss. These results demonstrate that Gpr126 is essential in the trophoblast lineage for the promotion of spiral artery remodeling during placental development. We propose that heart abnormalities in *Gpr126* mutants are secondary to placental defects, reflecting the intimate relationship between the placenta and fetal heart. Research into therapies aimed at modulating aGPCR signaling must consider potential deleterious effects in pregnancy.

## RESULTS

### *Gpr126*^Δ*7/*Δ*7*^-null mutants show defective cardiac development and die in embryogenesis

**Fig. 1.**
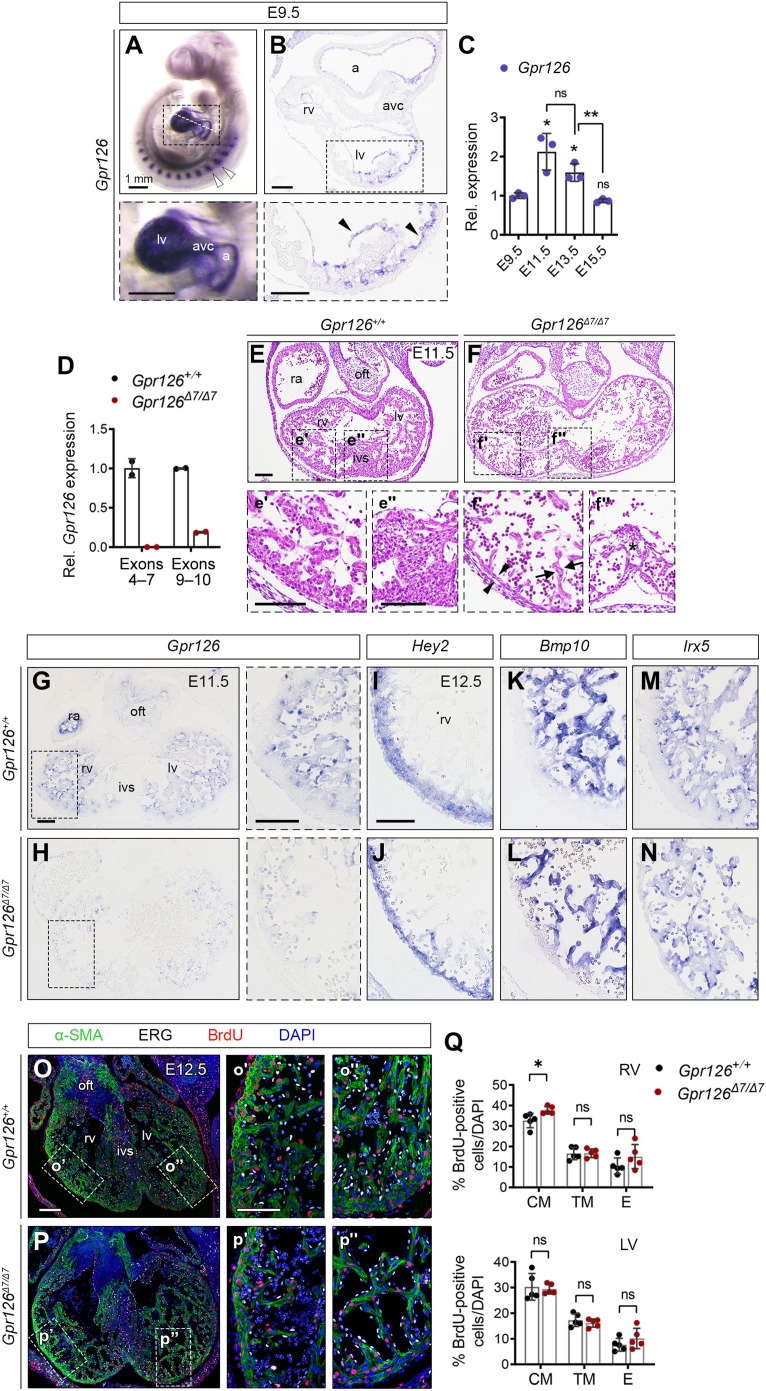
Defective chamber development in *Gpr126*^Δ*7/*Δ*7*^ mutant embryos is not associated with altered patterning or proliferation. (**A**) E9.5 WT embryo. Whole-mount in situ hybridization (WISH) showing *Gpr126* mRNA in somites (arrowheads) and heart (bottom). (**B**) Transverse heart sections. Bottom: Arrowheads indicate endocardial *Gpr126* expression. (**C**) Quantitative reverse transcription polymerase chain reaction (qRT-PCR): *Gpr126* transcription (relative to *Gapdh*) in embryonic WT hearts. Data are means ± SD (*n* = 3 pools of three hearts per pool per stage). Relative values normalized to E9.5. Statistics: Unpaired Student’s *t* test (ns, not significant; *P < 0.05; **P < 0.01). (**D**) qRT-PCR. *Gpr126* gene expression (relative to *Gapdh*) in *Gpr126 ^+/+^* and *Gpr126*^Δ*7/*Δ*7*^ E12.5 hearts, using primers spanning exons 4 to 7 and exons 9 and 10. Data are means ± SD (*n* = 2 hearts). (**E** to **F″**) Hematoxylin and eosin (H&E)–stained E11.5 *Gpr126^+/+^* (E to E″) and *Gpr126*^Δ*7/*Δ*7*^ heart sections (F to F″). Bottom panels: High magnifications of chamber (′) and septum (″). Note compact myocardium (arrowheads) and trabeculae (arrows) thinning and poorly cellularized ventricular septum (asterisk) in the mutant (F′ and F″). (**G** and **H**) *Gpr126* ISH, E11.5 *Gpr126^+/+^* (G) and *Gpr126*^Δ*7/*Δ*7*^ (H) heart sections. Right: Higher magnifications. (**I** to **N**) ISH of compact myocardium [*Hey2* (I and J)], trabecular myocardium [*Bmp10* (K and L)], and chamber endocardium [*Irx5* (M and N)] markers in E12.5 *Gpr126^+/+^* and *Gpr126*^Δ*7/*Δ*7*^ hearts. (**O** to **P″**) BrdU immunostaining (red) of E12.5 *Gpr126^+/+^* (O to O″) and *Gpr126*^Δ*7/*Δ*7*^ (P to P″) hearts. α-Smooth muscle actin (α-SMA) (green) counterstains the myocardium, ERG (white) counterstains the endocardium, and 4′,6-diamidino-2-phenylindole (DAPI) (blue) counterstains the nuclei. The panels (′ and ″) show high magnifications of boxed areas in (O) and (P). (**Q**) BrdU-positive nuclei quantification as a percentage of total nuclei (DAPI^+^) in the compact (CM), trabecular myocardium (TM), and endocardium (E) of E12.5 *Gpr126^+/+^* and *Gpr126*^Δ*7/*Δ*7*^ hearts. Data are means ± SD. Statistics: Unpaired Student’s *t* test. Scale bars, 100 μm (low magnification) and 50 μm (high magnification). a, atrium; avc, atrioventricular canal; ivs, interventricular septum; la, left atrium; lv, left ventricle; oft, outflow tract; ra, right atrium; rv, right ventricle.

In situ hybridization (ISH) showed *Gpr126* expression in the somites and heart of E9.5 mouse embryos (Fig. 1, A and B). Cardiac *Gpr126* expression was confined to the chamber endocardium throughout development (fig. S1, A to C). Real-time quantitative polymerase chain reaction (qPCR) revealed significant enrichment in E11.5 hearts (Fig. 1C). To study whether Gpr126 is required for heart development, we generated a *Gpr126-*null allele using CRISPR-Cas9 genome editing technology (see Methods). Computational analysis of exon 7 deletion predicted a translational frameshift to generate a premature termination codon (PTC) in exon 8, thus preventing translation of all Gpr126 isoforms (fig. S2, A and B). *Gpr126* transcript levels were abrogated in homozygous *Gpr126*^Δ*7/*Δ*7*^ mutants (Fig. 1D), indicating that the *Gpr126*^Δ*7*^ mutation is a null allele. Intercrosses of heterozygous *Gpr126*^Δ*7/+*^ animals yielded no viable homozygous mutant offspring, and homozygous *Gpr126*^Δ*7/*Δ*7*^ mutants died predominantly at E13.5 (table S1, sheet 1). These results are consistent with previous reports showing that Gpr126 is essential for embryonic progression (*5*, *7*). Histological analysis of E11.5 embryos revealed cardiac defects in *Gpr126*^Δ*7/*Δ*7*^ mutant hearts, with dilated ventricles (Fig. 1, E and F), thin compact myocardium and trabeculae (Fig. 1, E′ and F), and poorly developed interventricular septa (Fig. 1, E″ and F″). The atrioventricular canal and outflow tract (OFT) cushions were normal, suggesting that cardiac epithelial-to-mesenchyme transition was unaffected. The *Gpr126*^Δ*7/*Δ*7*^ mutant phenotype thus represents a true Gpr126 loss of function because it recapitulates the cardiac abnormalities described for the various *Gpr126* knockout mouse models (*5*, *7*, *15*). ISH confirmed the abrogation of endocardial *Gpr126* expression in E11.5 *Gpr126*^Δ*7/*Δ*7*^ hearts (Fig. 1, G and H). However, there was no detectable effect on the cardiac transcription of *Hey2* (*16*–*18*), *Bmp10* (*19*), and *Irx5* (*20*), which delineate the compact and trabecular myocardium and the ventricular endocardium (Fig. 1, I to N). We further assessed ventricular maturation in E12.5 hearts. ISH of the glycolytic genes lactate dehydrogenase a (*Ldha*) and pyruvate dehydrogenase kinase I (*Pdk1*) revealed similar chamber expression in control and *Gpr126*^Δ*7/*Δ*7*^ mutant hearts (fig. S3, A to D). The glucose transporter Glut1 was similarly unaffected (fig. S3, E and F). Hypoxia drives glycolysis in the developing heart (*21*), and hypoxyprobe immunoreactivity was restricted to the cushion-associated myocardium of the OFT in both E12.5 control and *Gpr126*^Δ*7/*Δ*7*^ hearts (fig. S3, G and H). We also detected no effect on cellular proliferation in E12.5 mutant hearts (Fig. 1, O to Q). These results indicated that chamber patterning, cardiac energy metabolism, hypoxia, and cellular proliferation were unaffected and thus were not the primary cause of defective ventricular wall development and death in *Gpr126*^Δ*7/*Δ*7*^ mutants.

### Gene profiling of *Gpr126*^Δ*7/*Δ*7*^ hearts

To identify the molecular targets downstream of Gpr126 in the heart, we profiled E12.5 *Gpr126^+/+^* and *Gpr126*^Δ*7/*Δ*7*^ mutant ventricles by RNA sequencing (RNA-seq) (fig. S4A). We identified 156 differentially expressed genes (DEGs), 77 of which were up-regulated and 79 down-regulated (adjusted *P* < 0.05; fig. S4B and table S2). Gene Ontology (GO) analysis revealed the overrepresentation of “vasculature development,” “cell migration,” and “angiogenesis” terms for the down-regulated genes (fig. S4C, top, and table S3), whereas for the up-regulated genes, the most enriched functions were “cellular response to amino acid stimulus,” “skeletal system development,” “cell adhesion,” and “negative regulation of cell signaling” (fig. S4C and table S3). We detected expression of a subset of down-regulated genes associated with endothelial functions in the embryonic endocardium (*Tmem100*, *Ednrb*, *Stab1*, *Eng*, *Kdr*, *Plxd1*, *Adamts1*, and *Tie1*) (fig. S4D and table S3). Moreover, we detected cardiac up-regulation of two G protein signaling regulators (*Rgs*)—*Rgs2* and *Rgs16—*that participate in the termination of GPCR signaling (fig. S4D and table S2) (*22*). ISH confirmed attenuated expression of *EdnrB* and *Eng* in the endocardium of *Gpr126*^Δ*7/*Δ*7*^ mutants (fig. S4, E to H), in line with the RNA-seq data (fig. S4D and table S2). These results thus suggest that global *Gpr126* deletion affects a set of genes expressed in the embryonic endocardium.

### Conditional *Gpr126* inactivation in the endocardium allows survival

**Fig. 2.**
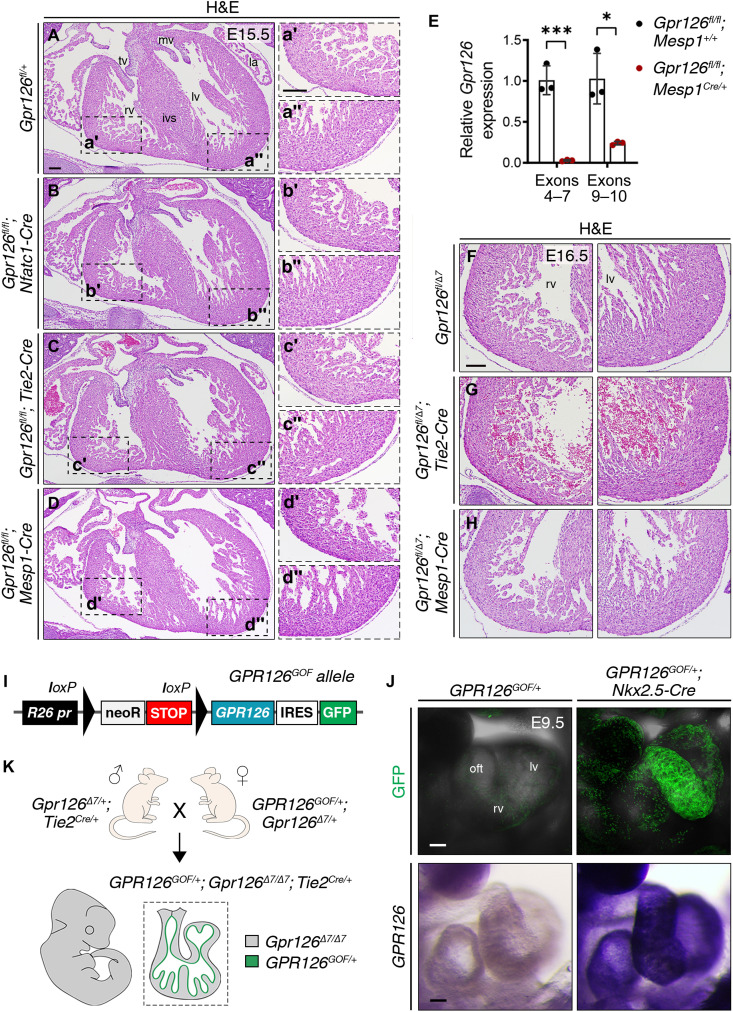
Conditional *Gpr126* deletion in either the endocardium or all cardiac tissues allows embryonic survival. (**A** to **D″**) H&E staining of transverse heart sections from WT (A to A″), *Gpr126^fl/fl^;Nfatc1^Cre/+^* (B to B″), *Gpr126^fl/fl^;Tie2^Cre/+^* (C to C″), and *Gpr126^fl/fl^;Mesp1^Cre/+^* (D to D″) embryos at E15.5. Magnified views of the right ventricle are shown in (′) and of the left ventricle in (″). (**E**) qRT-PCR analysis showing relative *Gpr126* gene expression (to *Gapdh*) in *Gpr126^fl/fl^;Mesp1^+/+^* and *Gpr126^fl/fl^;Mesp1^Cre/+^* embryonic hearts at E12.5 using primers spanning exons 4 to 7 and exons 9 and 10. Data are means ± SD (*n* = 3 embryonic hearts); *t* test, *P < 0.05 and ***P < 0.001. (**F** to **H**) H&E staining of transverse heart sections from WT *Gpr126*^*fl/*Δ*7*^ (F), *Gpr126*^*fl/*Δ*7*^*;Tie2^Cre/+^* (G), and *Gpr126*^*fl/*Δ*7*^*;Mesp1^Cre/+^* (H) embryos at E16.5. Left panels: Right ventricle; Right panels: Left ventricle. (**I**) Schematic of the conditional gain-of-function *R26-GPR126^GOF^* construct. *GPR126* expression is activated upon the Cre-mediated excision of the *NeoR-STOP* cassette. (**J**) Top panels: Confocal images showing eGFP expression in E9.5 *GPR126^GOF/+^* and *GPR126^GOF/+^;Nkx2.5^Cre/+^* hearts. Bottom panels: WISH showing *GPR126* expression in E9.5 *GPR126^GOF/+^* and *GPR126^GOF/+^;Nkx2.5^Cre/+^* hearts. (**K**) Representation of *GPR126^GOF/+^;Gpr126*^Δ*7/*Δ*7*^*;Tie2^Cre/+^* embryos from crosses of *Gpr126*^Δ*7/+*^*;Tie2^Cre/+^* males with *GPR126^GOF/+^;Gpr126*^Δ*7/+*^ females. Scale bars, 100 μm. GFP, green fluorescent protein; IRES, internal ribosome entry site; mv, mitral valve; neoR, neomycin resistance gene; *R26 pr*, *Rosa26* promoter; tv, tricuspid valve.

We next generated a conditional *Gpr126^flox^* line, in which Cre-mediated excision of exon 7 results in the same loss of coding sequence as in the global *Gpr126*^Δ*7*^ knockout mice (fig. S2C). We then crossed conditional *Gpr126^flox^* mice with the endocardial-specific driver line *Nfatc1^panCre^*, in which the Cre recombinase is active throughout the endocardium from E8.5 (*23*). Unexpectedly, *Gpr126^fl/fl^;Nfatc1^panCre^* mutant embryos were viable throughout embryogenesis (table S1, sheet 2) unlike *Gpr126*^Δ*7*^ knockout mice (table S1, sheet 1) and showed no heart abnormalities (Fig. 2, A to B″). Viable embryos also resulted from crosses of conditional *Gpr126^flox^* mice with the pan-endothelial driver line *Tie2^Cre^*, active from E7.5 (*24*) (table S1, sheet 3); moreover, *Gpr126^fl/fl^;Tie2^Cre^* mutant embryos had normal cardiac morphology (Fig. 2, A to A″ and C to C″) and reached adulthood. To exclude the possibility that Gpr126 plays an earlier role in cardiac mesoderm progenitors, we crossed *Gpr126^flox^* mice with the *Mesp1^Cre^* line, in which Cre recombinase is active at the onset of gastrulation (E6.5) in the nascent mesoderm that later gives rise to all cardiac cell lineages (*25*). As with the other crosses, *Gpr126^fl/fl^;Mesp1^Cre^* mutants were viable (table S1, sheet 4) and did not show any obvious heart phenotype (Fig. 2, D to D″). qRT-PCR analysis in *Gpr126^fl/fl^*;*Mesp1^Cre/+^* embryonic hearts verified Cre recombination efficiency in *Gpr126^flox^* mutants, with *Gpr126* transcript levels undetectable in E12.5 mutant hearts, similar to global *Gpr126*^Δ*7/*Δ*7*^ (Fig. 2E). Last, to generate the most extreme conditional loss-of-function situation, we combined the *Gpr126* conditional (*Gpr126^flox^*) and standard (*Gpr126*^Δ*7*^) alleles in trans configuration. Crosses of homozygous *Gpr126^fl/fl^* mice with double heterozygotes *Gpr126*^Δ*7/+*^*;Tie2^Cre/+^* and *Gpr126*^Δ*7/+*^*;Mesp1-Cre/+* mice yielded trans heterozygous *Gpr126*^*fl/*Δ*7*^*;Tie2^Cre/+^* (table S1, sheet 5) and *Gpr126*^*fl/*Δ*7*^*;Mesp1^Cre/+^* (table S1, sheet 6) offspring, respectively. Neither of these mutants showed evidence of embryonic lethality or cardiac defects (Fig. 2, F to H).

cAMP modulates several cardiac functions, including heart rate and contractility (*26*). Because cAMP/cAMP-dependent protein kinase (PKA) are downstream mediators of *Gpr126* signaling (*9*, *27*), we asked whether Gpr126 was required for adult cardiac electrical activity or homeostasis. Electrocardiograms (ECGs) obtained from 31- to 36-week-old male and female *Gpr126^fl/fl^;Tie2^Cre/+^* mice revealed unaltered values for all measured ECG parameters compared with sex-matched controls (fig. S5A). Cardiac function measured by ultrasound was similarly normal (fig. S5B). Moreover, *Gpr126^fl/fl^;Tie2^Cre/+^* mice showed no differences from controls in heart size determined as the heart weight–to–tibia length ratio (fig. S5C) or in cardiac morphology or collagen deposition, assessed by Masson’s trichrome staining on histological sections (fig. S5, D to E″). Collectively, these data demonstrated that endothelial *Gpr126* function is dispensable for cardiac development and adult heart homeostasis and that the embryonic demise of *Gpr126*^Δ*7*^ homozygous mutants is therefore due to an essential role of Gpr126 in noncardiac tissues.

### Conditional *GPR126* expression in the endocardium does not rescue *Gpr126*^Δ*7*^ embryonic lethality

To confirm the noninvolvement of cardiac Gpr126 signaling in embryonic progression, we generated a transgenic line conditionally overexpressing human *GPR126* complementary DNA (cDNA) under the control of the ubiquitous *Rosa26* promoter (*GPR126^GOF^*; see Methods) (Fig. 2I and fig. S6) (*28*). *Nkx2.5^Cre^*-mediated (*29*) removal of the *NeoSTOP* sequences resulted in *Rosa26*-driven *GPR126-EGFP* expression in the heart. Analysis of E9.5 transgenic *GPR126^GOF/+^;Nkx2.5^Cre/+^* embryos revealed widespread cardiac green fluorescent protein (GFP) expression (Fig. 2J, top), and whole-mount ISH (WISH) revealed *GPR126* mRNA expression throughout the heart (Fig. 2J, bottom). Similarly, *GPR126^GOF/+^;Tie2^Cre/+^* embryos overexpressing *GPR126* in the endothelium and endocardium showed normal cardiac morphology (fig. S7, A to F″) and were viable (table S1, sheet 7). We then generated embryos in which endogenous *Gpr126* is globally disrupted and *GPR126* expression is confined to the *Tie2-Cre* lineage (*GPR126^GOF/+^;Gpr126*^Δ*7/*Δ*7*^;*Tie2^Cre/+^*; Fig. 2K). These embryos died at the same gestational stages as *Gpr126*^Δ*7/*Δ*7*^ mutants (table S1, sheet 8), indicating that forced GPR126 expression in the endocardium does not rescue the embryonic lethality caused by global Gpr126 loss.

### The Gpr126 CUB and PTX domains are dispensable in the heart but essential for PNS development

The Gpr126 NTF has been proposed to be required for heart development in mice and zebrafish (*5*). The NTF contains five domains: the complement C1r/C1s, Uegf, Bmp1 (CUB) domain; the pentraxin (PTX) domain; the hormone-binding domain (HBD); the GAIN domain (*30*); and the recently described sperm protein, enterokinase, and agrin (SEA) domain (*31*) (fig. S2D). The CUB domain contains a conserved calcium-binding site necessary for a close ECR conformation and is critical for zebrafish gpr126 function (*5*). To determine whether the adhesive CUB and PTX domains in the NTF are necessary for heart development, we generated a *Gpr126* knockout mouse model with an in-frame deletion of exons 3 and 4 (*Gpr126*^Δ*3*,*4*^; fig. S2, E and F). This mutation is predicted to encode a shortened NTF lacking the CUB and PTX domains (fig. S2G). *Gpr126*^Δ*3*,*4/+*^ heterozygous mating yielded homozygous offspring in the expected Mendelian inheritance ratio during embryonic development and in live births (table S1, sheet 9). Histological analysis revealed normal heart morphology in *Gpr126*^Δ*3*,*4*^ homozygotes from E12.5 to postnatal day 2 (P2) (fig. S8, A to F). Nevertheless, the forelimbs and hindlimbs of *Gpr126*^Δ*3*,*4*^ homozygous mutant pups were severely affected. Mutants were slightly smaller in size than their control and heterozygous littermates and had joint contractures that impeded body balance and normal locomotion (fig. S8G). This limb phenotype has been linked to the requirement of *Gpr126* for Schwann cell myelination in mammals and zebrafish (*15*) and recapitulates the human disorder called AMC (*13*). Reverse transcription PCR (RT-PCR) with primers spanning exon 24 and the 3′ untranslated region (3′UTR) detected normal *Gpr126* mRNA levels in control, heterozygous, and homozygous mutant embryos by qPCR (fig. S8, H and I), suggesting that the mutants expressed a shorter Gpr126 protein lacking exons 3 and 4. Together, our results demonstrate that lack of the CUB and PTX domains within the Gpr126 NTF compromises PNS myelination but does not affect heart development in mice.

### Generation of zebrafish *gpr126* loss-of-function mutants

Our mouse results raised the intriguing possibility that the defective cardiac development and death of global *Gpr126* mutants were secondary to the loss of Gpr126 in another organ. The zebrafish has been widely used to study the role of *gpr126* in development. One report described a morpholino-based model in which the NTF of Gpr126 acts as a paracrine signal required for ventricular trabeculation (*5*). Paradoxically, despite the large number of *gpr126* zebrafish mutant lines analyzed, no heart phenotype has been described thus far (*3*, *4*, *8*, *31*, *32*). The phenotypic discrepancies observed between knockdown and knockout animals, along with our mouse results, prompted us to study the function of Gpr126 in zebrafish heart development.

We used CRISPR-Cas9 technology to generate two *gpr126* mutant alleles: *gpr126^bns341^* and *gpr126^bns342^* (see Methods). In the zebrafish *gpr126^bns341^* allele, a deletion of exons 7 and 8 leads to a frameshift and a PTC in exon 10 (fig. S9, A and B). Because mouse and zebrafish *Gpr126* are considered true orthologs (*7*, *33*), the similarity to our *Gpr126*^Δ*7*^ mouse line would illustrate the parallels in gene function between these animal models. The *gpr126^bns341^* mutation is predicted to truncate the protein between the linker region (encoded by exon 5) and the SEA domain (*31*) encoded by exons 7 to 10 (fig. S9C). In contrast, the *gpr126^bns342^* allele is a promoter-less allele (fig. S9, D and E).

*gpr126* is not duplicated in the zebrafish genome (*7*), and we searched for genes with high sequence similarity to identify potential compensatory paralogs or “adapting genes” (*34*), which might attenuate a potential cardiac phenotype of *gpr126* mutants (see Methods). In agreement with previous reports (*7*, *33*), the closest homologs that we found to zebrafish *gpr126* were *adgrg4b* (also known as *gpr112b*), *adgrg4a* (or *gpr112a*), and *adgrg2a* (or *gpr64a*) (table S4, sheet 1). The same *Gpr126* homologs (*Adgrg4*/*Gpr112* and *Adgrg2*/*Gpr64*) were found in mice (table S4, sheet 2), and the zebrafish genes are expressed during zebrafish development (*33*). Moreover, zebrafish Gpr112 has been reported to share more protein homology with Gpr126 than is the case for the mammalian proteins (*7*), suggesting that *gpr112* might be a candidate compensating gene for the loss of *gpr126*.

**Fig. 3.**
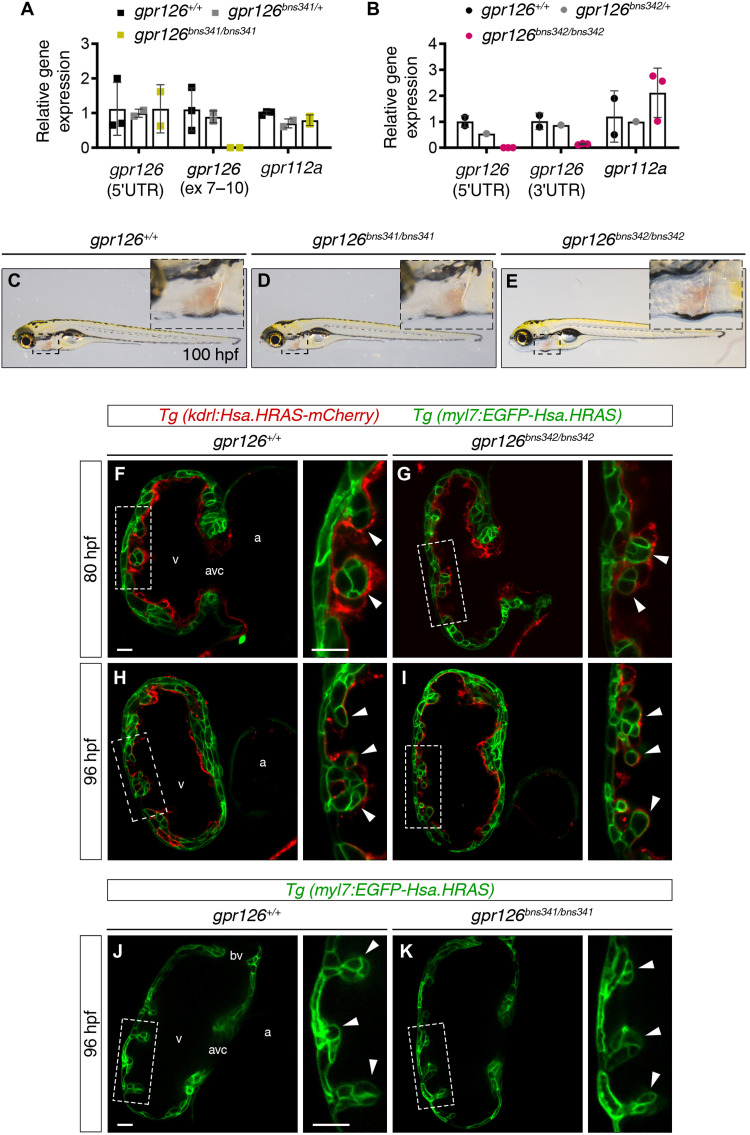
Trabeculation is unaffected in *gpr126^bns341^* and *gpr126^bns342^* zebrafish mutants. (**A** and **B**) RT-qPCR analysis of relative *gpr126* and *gpr112a* mRNA expression (to the housekeeping gene *rpl13a*) in WT zebrafish and heterozygous and homozygous *gpr126^bns341^* (A) and *gpr126^bns342^* (B) zebrafish mutants at 48 hours postfertilization (hpf). Data are means ± SD (*n* = 1, 2, or 3 pools of three hearts per pool). (**C** to **E**) Lateral views of 100 hpf *gpr126^+/+^* (E), *gpr126^bns341/bns341^* (D), and *gpr126^bns342/bns342^* (E) zebrafish larvae. Inset panels: High-magnification views of the heart (boxed area). (**F** to **I**) Mid-sagittal confocal sections of *gpr126^+/+^* and *gpr126^bns342/bns342^* zebrafish hearts at 80 hpf (F and G) and 96 hpf (H and I) in larvae derived from *gpr126^bns342/+^;Tg(kdrl:Hsa.HRAS-mCherry);Tg(myl7:EGFP-Hsa.HRAS)* incrosses. (**J** and **K**) Mid-sagittal confocal sections of *gpr126^+/+^* and *gpr126^bns341/bns341^* zebrafish hearts at 96 hpf in larvae derived from *gpr126^bns341/+^;Tg(myl7:EGFP-Hsa.HRAS)* incrosses. Endocardial and myocardial membranes in (F) to (K) are labeled red and green, respectively. Right panels: High-magnification views of the outer curvature of the ventricular wall corresponding to the boxed areas. Arrowheads indicate emerging trabeculae. Scale bars, 25 μm. bv, bulboventricular valve; v, ventricle.

We next determined the effect of the *gpr126^bns341^* and *gpr126^bns342^* mutations on gene transcription by examining *gpr126* and *gpr112a* mRNA levels at 73 hours postfertilization (hpf), when *gpr126* starts to be expressed (*33*). RT-qPCR revealed similar *gpr126* transcript levels using 5′UTR primers in *gpr126^+/+^*, *gpr126^bns341/+^*, and *gpr126^bns341/bns341^* larvae and no transcripts of exons 7 to 10 in *gpr126^bns341/bns341^* larvae (Fig. 3A), while similar amounts of *gpr112a* mRNA were found in all three genotypes (Fig. 3A). These results suggest that although the *gpr126^bns341^* mutation generates a PTC in *gpr126* exon 10, it does not lead to mutant mRNA degradation, and up-regulation of *gpr112a* is not observed. It thus seems likely that the *gpr126^bns341^* mutant allele produces a truncated protein formed by the SP, CUB, PTX, and linker domains of the ECD (fig. S9C). In contrast, *gpr126* mRNA levels in *gpr126^bns347bns3422^* larvae are very substantially reduced (Fig. 3B), and *gpr112a* levels remain unchanged compared with control siblings (Fig. 3B).

### Zebrafish *gpr126^bns341^* and *gpr126^bns342^* mutants display WT-like cardiac trabeculation

Zebrafish *gpr126* plays an essential role in inner ear development, and mutants can be easily identified by a swollen ear phenotype caused by abnormal semicircular canal morphogenesis (*3*, *11*, *31*). As reported in other zebrafish *gpr126* mutants (*11*), *gpr126^bns341^* and *gpr126^bns342^* homozygous mutants display an enlarged ear phenotype at 5 days postfertilization (dpf) (fig. S10, A to E), indicating Gpr126 loss of function. In the zebrafish heart, *gpr126* is expressed at 48 hpf, before the onset of trabeculation (*5*, *14*). Homozygous *gpr126^bns341^* and *gpr126^bns342^* fish reached adulthood and exhibited no gross developmental phenotype (not shown). Overall heart morphology also appeared unaffected in these mutants, and there was no evidence of pericardial edema at 100 hpf, when trabeculation is well advanced (Fig. 3, C to E). To examine ventricular development in more detail, we bred *gpr126^bns342/+^* fish into the *Tg(kdrl:Hsa.HRAS-mCherry)*;*Tg(myl7:EGFP-Hsa.HRAS)* background (*35*, *36*) to label the endocardial and myocardial membranes red and green, respectively. Trabeculae start to form in zebrafish around 60 hpf and become evident by 72 hpf (*37*). Confocal imaging at 80 hpf revealed no obvious differences between mutant and control siblings (Fig. 3, F and G). Ventricles in the mutant fish contained delaminated cardiomyocytes forming nascent trabecular ridges, thus indicating normal trabeculation onset (Fig. 3, F and G). No trabeculation defects were observed at 96 hpf, with trabecular projections present throughout the luminal side of the outer ventricular curvature, similarly to control siblings (Fig. 3, H and I). The absence of a heart phenotype in *gpr126^bns342^* mutants, which exhibit strongly reduced *gpr126* transcriptional activity, predicted similar results in *gpr126^bns341^* larvae, which presumably encode a truncated Gpr126 protein. To test this prediction, we bred the *gpr126^bns341^* mutation into the *Tg(myl7:EGFP-Hsa.HRAS)* transgenic line. Confocal microscopy analysis of homozygous *gpr126^bns341^* hearts revealed extensive trabeculation at 96 hpf, as in control siblings (Fig. 3, J and K). These data thus suggest that *gpr126* signaling is not required for trabeculation in zebrafish.

### *Gpr126* is expressed in the spongiotrophoblast layer, TGCs, and endothelium of the maternal spiral arteries in the mouse placenta

**Fig. 4.**
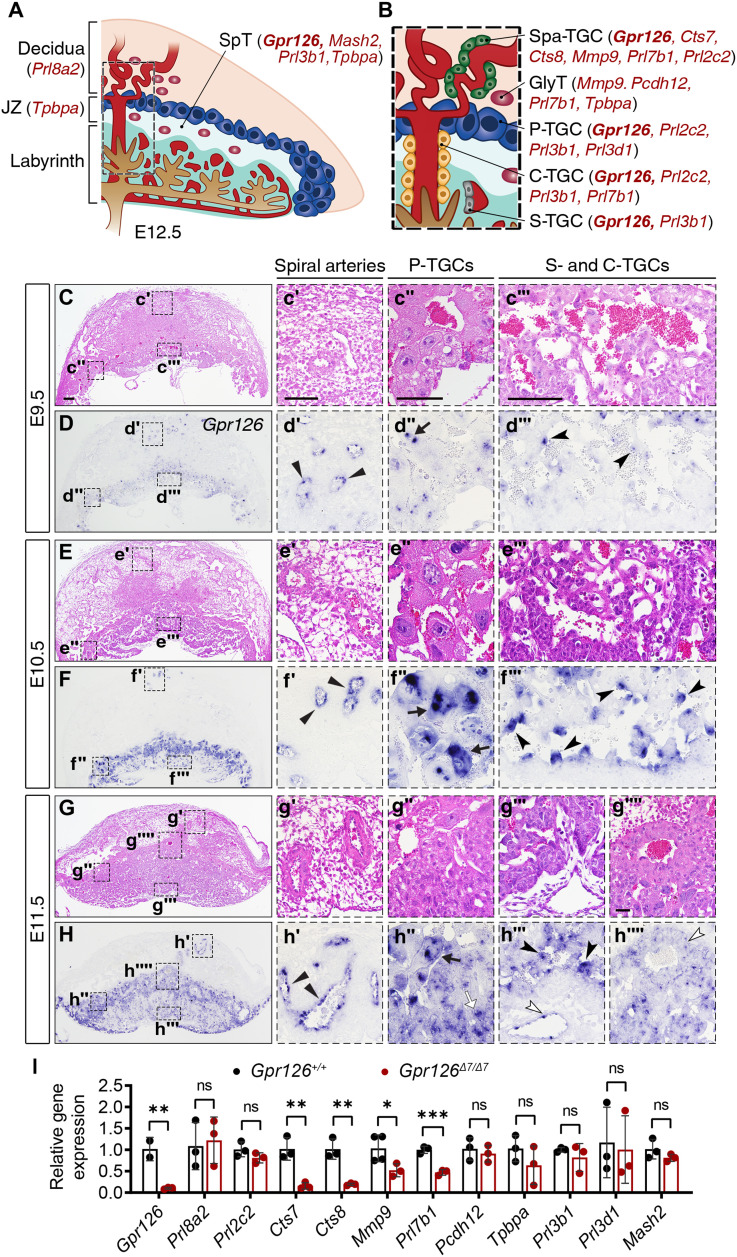
*Gpr126* is expressed in the mouse placenta and controls the expression of Spa-TGC markers. (**A** and **B**) Cartoon illustrating the main tissue layers (A) and cell types (B) of a WT E12.5 mouse placenta and key marker genes that are expressed in each cell type. Note that family members of the trophoblast giant cell–expressed *Prl* (prolactin/placental lactogen/prolactin-like protein) genes are also known as follows: *Prl3d1 = Pl1*, *Prl3b1 = Pl2*, *Prl2c2 = Plf*, *Prl8a2 = Dtprp*, *Prl7b1 = PlpN*. (**C** to **H⁗**) H&E staining (C to C‴, E to E‴, and G to G⁗) and ISH analysis (D to D‴, F to F‴, and H to H⁗) of *Gpr126* on sagittal sections of E9.5, E10.5, and E11.5 WT placentas. Right panels (′, ″, ‴, and ⁗): High-magnification views of the boxed areas, showing the spiral artery-associated trophoblast giant cells (Spa-TGCs) (′, with gene expression marked with black triangular arrowheads), parietal TGCs (P-TGCs) (″, with gene expression marked with black arrows, except for the white arrow in h’’ that points to the SpT), sinusoidal TGCs (S-TGCs) (‴, with gene expression marked with black notched arrowheads), and canal TGCs (C-TGCs) (⁗, with gene expression marked with white notched arrowheads). Scale bars, 200 μm (low magnification); 50 μm (insets). (**I**) qRT-PCR showing relative gene expression of key markers shown in (A and B) in E11.5 *Gpr126^+/+^* and *Gpr126*^Δ*7/*Δ*7*^ placentas. β-Actin was used as a housekeeping gene. Data are means ± SD (*n* = 3 WT and 3 mutant placentas and 4 WT and 4 mutant placentas for *Mmp9* expression analysis). Statistical significance was determined by unpaired Student’s *t* test (*P < 0.05; **P < 0.01; ***P < 0.001). GlyT, Glycogen trophoblast cell; JZ, junctional zone; SpT, spongiotrophoblast.

*Gpr126* is expressed in the mouse placenta (*7*). The placenta is critical for fetal survival and growth because it forms the interface between the maternal and fetal circulation, facilitating metabolic and gas exchange, as well as fetal waste disposal. The placenta also produces hormones that alter maternal physiology during pregnancy and forms a barrier that protects the fetus against the maternal immune system (*38*). In rodents, the placenta consists of three broad zones (Fig. 4A). The maternal decidua on the outside is devoid of trophoblast cells until mid-gestation, when fetal trophoblast cells invade the decidua and reach the spiral arteries, replacing the endothelium to promote the transition from endothelial cell–lined to trophoblast cell–lined maternal blood spaces (Fig. 4A) (*39*). The next two layers are of fetal origin. The junctional zone (JZ) consists of spongiotrophoblast (SpT) cells and a layer of TGCs that line the implantation site (Fig. 4A). The innermost layer is the labyrinth, a tortuous network of maternal and fetal blood spaces that is the site of nutrient, gas, and waste exchange between the fetal and maternal circulations (Fig. 4A) (*40*). The mouse placenta is mature when the fetal-maternal interface is evidenced by the presence of maternal blood in the labyrinth at E12.5. Human and rodent placentas are hemochorial, so that trophoblast cells and not endothelial cells line the maternal vasculature within the placenta. In mice, the four different TGC subtypes are named according to their location with respect to the maternal circulation (*40*, *41*). There are spiral artery–associated TGCs (SpA-TGCs) that invade into the spiral arteries of the decidua and displace the maternal endothelial cells, canal-associated TGCs (C-TGCs) that line the maternal blood canals as they pass through the JZ to the bottom of the labyrinth, sinusoidal-associated TGCs (S-TCGs) that sit within the maternal blood spaces of the labyrinth, and parietal TGCs (P-TGCs) that surround lacunae of blood leading into the uterine veins and line the implantation site (Fig. 4B) (*42*). Analysis of *Gpr126* expression by ISH during placental development revealed *Gpr126* at E9.5 in the maternal spiral arteries within the decidua (Fig. 4, B to D″), in the P-TGCs that delineate the implantation site (Fig. 4, C″ and D″), and in the S-TGCs lining maternal blood sinusoids within the labyrinth layer of the placenta (Fig. 4, C‴ and D‴). At E10.5, we found a similar pattern but stronger expression (Fig. 4, E to F‴). At E11.5, *Gpr126* expression was maintained in these cells (Fig. 4, G to H″) and was also found in the SpT (Fig. 4, G″ and H″), in the C-TGCs surrounding maternal canal spaces within the labyrinth at the base of the placenta (Fig. 4, G‴ and H‴) and extending through the JZ (Fig. 4, G‴ and H‴). To identify the cell types in which *Gpr126* is expressed, we compared *Gpr126* expression with that of the placental trophoblast marker *Prl2c2* by ISH. At E10.5, *Gpr126* mRNA was detected in P-TGCs (fig. S11, A to A″) and invasive trophoblasts (fig. S11A″) similarly to *Prl2c2* (fig. S11, B to B″). However, distal spiral arteries were *Gpr126* positive (fig. S11A‴) and negative for *Prl2c2* expression (fig. S11B‴), indicating that *Gpr126* is expressed not only in Spa-TGCs but also in the maternal endothelium. At E11.5, *Gpr126* expression in the maternal-fetal interface was compared to that of endogenous alkaline phosphatase (ALP) activity in the labyrinth (fig. S11, C and D). *Gpr126* is strongly expressed in trophoblasts facing maternal blood sinuses, likely being S-TGC (fig. S11, C′ and C″), while ALP is localized to the syncytiotrophoblast layer underneath (fig. S11, D′ and D″). Thus, *Gpr126* is expressed in the SpT layer, in the four TGC subtypes of the mouse placenta, and in the endothelium of the maternal spiral arteries, suggesting a potential association between placental *Gpr126* and embryonic development.

### *Gpr126*^Δ*7/*Δ*7*^ mutants show down-regulation of placental cathepsins and the preeclampsia marker *Mmp9*

*Gpr126* is expressed in the four TGC subtypes and the SpT, which can be distinguished by their spatial localization and gene expression profiles (Fig. 4, A and B) (*41*). To study the requirement of Gpr126 for trophoblast lineage differentiation, we conducted a qRT-PCR gene expression analysis of several hormones and proteases involved in maternal adaptation to pregnancy. First, we confirmed the abrogation of *Gpr126* transcription in *Gpr126*^Δ*7/*Δ*7*^ mutant placentas (Fig. 4I). Among the prolactin-like (*Prl*) gene family members expressed in different placental cell types (*43*) (Fig. 4, A and B), only *Prl7b1* was down-regulated in *Gpr126*^Δ*7/*Δ*7*^ placentas (Fig. 4I). *Prl7b1* is expressed by invasive trophoblast cells [Spa-TGCs, C-TGCs, and Glycogen trophoblast cell (GLyT)] (Fig. 4B) (*43*). We also examined extracellular proteases, such as placental cathepsins, matrix metalloproteases (MMPs), and a disintegrin and metalloproteinases (ADAMs), that confer invasive and vascular remodeling capacity on fetal trophoblasts. *Cts7* and *Cts8*, which are expressed in Spa-TGCs (Fig. 4B), were severely reduced in *Gpr126*^Δ*7/*Δ*7*^ placentas (Fig. 4I). Mmp2 and Mmp9 are the most abundant gelatinases expressed during trophoblast invasion in humans, and reduced expression of Mmp9 is associated with preeclampsia in mice and humans (*44*, *45*). *Mmp2* was expressed at very low levels at E11.5 (not shown), and *Mmp9* was down-regulated in *Gpr126*^Δ*7/*Δ*7*^ mutant placentas (Fig. 4I), suggesting defective remodeling of maternal spiral arteries. *Adam17*, whose expression is induced by oxidative stress in preeclamptic placentas (*46*), was normally expressed (fig. S12A). Invasive trophoblast and decidua cells also express protease inhibitors, like *Timp1* and *Cst3*, which are tissue inhibitors of metalloproteinases and cathepsin inhibitors, respectively. Although preeclampsia associates with high expression of these genes, they were normally expressed in mutant placentas (fig. S12A). The JZ contains the progenitors for invasive trophoblasts (*47*), and examination of the SpT marker *Mash2*/*Ascl2*, the GlyT marker *Pcdh12*, and the JZ marker *Tpbpa* (Fig. 4, A and B) revealed no significant changes in mutant placentas (Fig. 4I). We next performed an ISH analysis of *Prl2c2*, *Mash2*, *Pcdh12*, and *Tpbpa* transcripts on sections of E11.5 WT and *Gpr126*^Δ*7/*Δ*7*^ placenta. *Prl2c2* expression in invading SpA-TGCs revealed thinner spiral arteries in the decidua close to the JZ in *Gpr126*^Δ*7/*Δ*7*^ placentas, whereas expression in the C-TGCs appeared to be unaffected (fig. S12, B and C). The JZ marker *Tpbpa* (fig. S12, D and E) and the SpT marker *Mash2* (fig. S12, F and G) revealed normal structures in mutant placentas. At E11.5, *Mash2* was also transcribed in the labyrinthine trophoblasts (fig. S12, F and G). Wrapping of *Pcdh12*-positive GlyT cells around maternal spiral arteries was impaired in *Gpr126*^Δ*7/*Δ*7*^ placentas (fig. S12, H and I). As expected, *Gpr126* expression was abrogated in *Gpr126*^Δ*7/*Δ*7*^ mutant placentas (fig. S12, J and K), in agreement with the qRT-PCR data (Fig. 4I). *Gpr126*^Δ*7/*Δ*7*^placentas show down-regulation of *Prl7b1*, *Mmp9*, and the placental cathepsins *Cts7* and *Cts8*. Together, these observations suggest that Gpr126 supports the invasive behavior of TGCs and the remodeling of the uterine vasculature by controlling the expression of extracellular proteases.

### Placental *Gpr126* is essential for embryonic survival

To determine the requirement of *Gpr126* for placental development and embryonic survival, we crossed mice bearing the conditional *Gpr126^flox^* allele with the *Sox2^Cre^* driver line (*48*). Paternal inheritance of *Sox2^Cre^* drives recombination in epiblast cells and in the extraembryonic mesoderm that forms the fetal vasculature in the placental labyrinth, but not in trophoblast-derived cells or in the visceral yolk sac endoderm (*48*, *49*). In contrast, if *Sox2^Cre^* is transmitted from the mother, then Cre is active in both embryonic and extraembryonic tissues due to female germline expression of Sox2 (*50*). Therefore, progeny arising from a *Sox2^Cre^* transgenic female will have Cre activity, regardless of genotype.

**Fig. 5.**
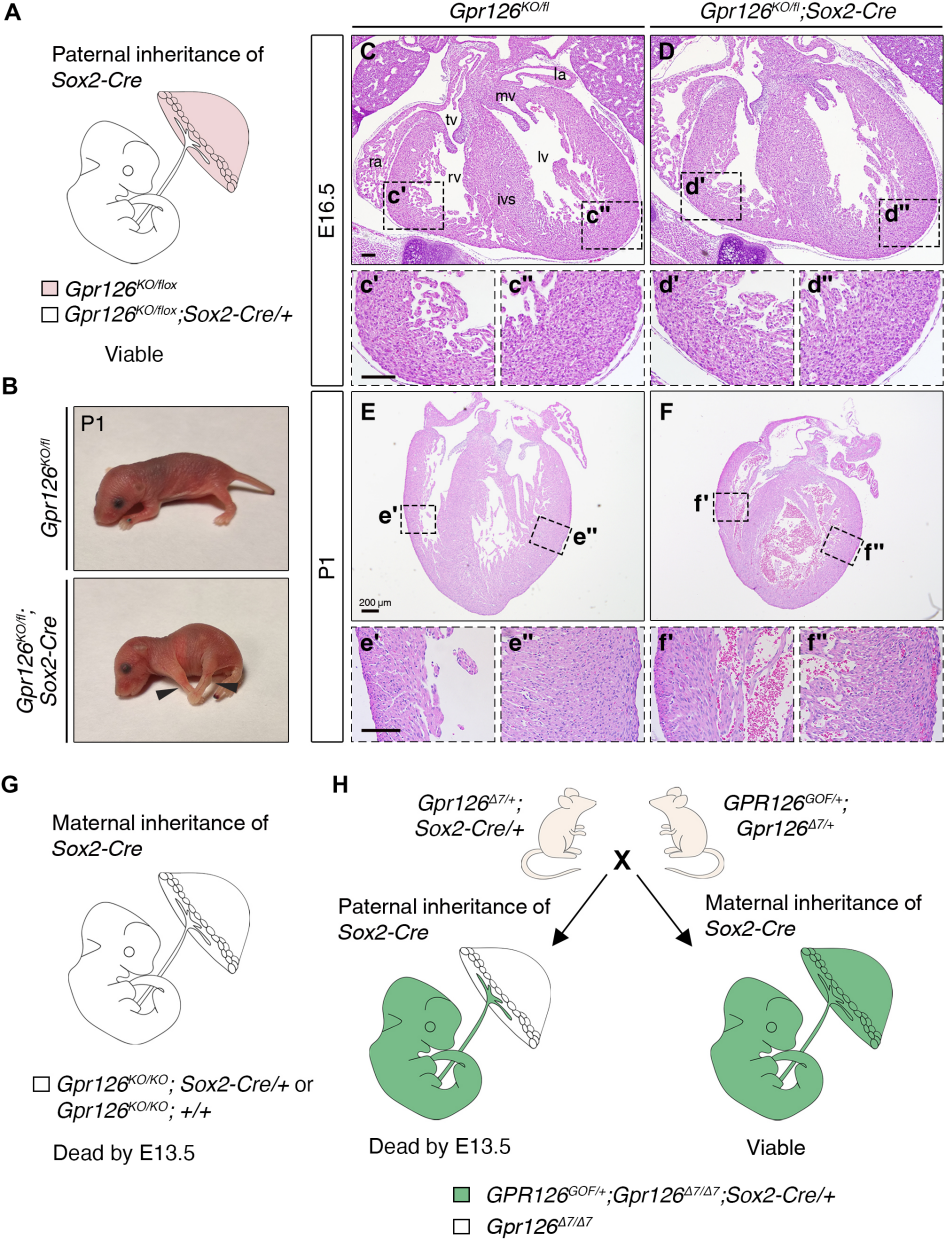
Dissection of embryonic and trophoblast-induced effects of *Gpr126* mutation. (**A**) Representation of the embryonic and extraembryonic genetic constitution of *Gpr126^fl/fl^;Sox2^Cre^* embryos when the *Sox2^Cre^* transgene is inherited paternally. Zygotic *Sox2^Cre^* expression deletes *Gpr126* in the embryo proper and in the embryonic component of the placenta (white). *Gpr126* is not inactivated in the maternal component of the placenta (pink). (**B**) Images of *Gpr126^KO/fl^* and *Gpr126^KO/fl^;Sox2^Cre^* mouse pups at P1. Arrowheads point to joint contractures in the forelimbs and hindlimbs of the mutants. (**C to F″**) H&E staining of transverse heart sections from *Gpr126^KO/fl^* and *Gpr126^KO/fl^;Sox2^Cre^* mutant embryos at E16.5 (C to D″) and P1 (E to F″). Bottom panels: High-magnification views of the right ventricle (′) and left ventricle (″) corresponding to the boxed areas in the main panels. Scale bars, 100 μm. (**G**) Representation of the embryonic and extraembryonic genetic constitution of *Gpr126^fl/fl^;Sox2^Cre^* embryos when the *Sox2^Cre^* transgene is inherited maternally. Note *Gpr126* inactivation in both the embryo proper and in the placenta. (**H**) Breeding strategy used to obtain *GPR126^GOF/+^;Gpr126*^Δ*7/*Δ*7*^*;Sox2^Cre/+^* embryos. The parental origin of the *Cre* transgene determines whether forced *GPR126^GOF^* expression (green) will take place only in the epiblast (paternal inheritance) or will be widespread in embryonic and extraembryonic tissues (maternal inheritance).

We first introduced the *Sox2^Cre^* transgene paternally by crossing *Gpr126^fl/+^;Sox2^Cre/+^* males with *Gpr126^fl/fl^* females to produce embryonic-specific deletion of *Gpr126* while the trophoblast tissue was heterozygous for *Gpr126* (Fig. 5A). If defective placentation was the cause of *Gpr126*^Δ*7/*Δ*7*^ embryonic lethality, then we would expect survival of *Gpr126^KO/fl^;Sox2^Cre/+^* offspring after E13.5, a stage when the standard knockout is already dead. This was the case; 73% of *Gpr126^KO/fl^;Sox2^Cre/+^* mutants were viable beyond E13.5, and 64% survived to birth (table S1, sheet 10). However, these mutant pups were distinguishable by their smaller size and stiff joint contractures in the forelimbs and hindlimbs (Fig. 5B), resembling the phenotype of *Gpr126*^Δ*3*,*4/*Δ*3*,*4*^ mutants (fig. S8G). Histological analysis of mutant embryonic hearts at E16.5 revealed no morphological defects (Fig. 5, C to D″). Examination at postnatal stages (P1) also showed normal heart structure, but the hearts were smaller (Fig. 5, E to F″), likely because the mutant embryos were also substantially smaller overall. We next introduced the *Sox2^Cre^* transgene maternally, by crossing *Gpr126^fl/fl^* males with *Gpr126^fl/+^;Sox2^Cre/+^* females to generate animals lacking *Gpr126* in both embryonic and extraembryonic tissues, regardless of the presence of the *Cre* transgene (Fig. 5G) (*50*). In these crosses, the percentage of homozygous mutants (referred to as *Gpr126^KO/KO^*) was increased from 25 to 50%, and they phenocopied standard *Gpr126*^Δ*7/*Δ*7*^ mutant embryos and did not survive past E13.5 (table S1, sheet 11). These results indicate that Gpr126 is required for the development of the maternally derived extraembryonic tissues of the placenta. To support this finding, we investigated whether forced expression of *GPR126* in the epiblast, or in the epiblast and the placenta, could rescue the lethality of *Gpr126*^Δ*7/*Δ*7*^ mutants. For this, we introduced the *Sox2^Cre^* transgene paternally or maternally in crosses involving *R26-GPR126^GOF/GOF^;Gpr126*^Δ*7/+*^ and *Gpr126*^Δ*7/+*^*;Sox2^Cre/+^* animals to generate *R26-GPR126^GOF/+^;Gpr126*^Δ*7/*Δ*7*^*;Sox2^Cre/+^* embryos. Within the *Gpr126*^Δ*7/*Δ*7*^ mutant class, offspring inheriting *Sox2^Cre^* paternally would express *GPR126* only in the embryo, whereas maternal inheritance would allow *GPR126* expression also throughout the trophoblast-derived cells of the placenta (Fig. 5H). Paternal inheritance of *Sox2^Cre^* did not support survival of *R26-GPR126 ^GOF/+^;Gpr126* ^Δ*7/*Δ*7*^*;Sox2^Cre/+^* mutants beyond E13.5 (table S1, sheet 12), whereas maternal inheritance of *Sox2^Cre^* allowed survival of *R26-GPR126^GOF/+^;Gpr126*^Δ*7/*Δ*7*^*;Sox2^Cre/+^* pups to birth—regardless of the presence of the Cre transgene (table S1, sheet 13)—and prevented abnormal PNS-related phenotypes (not shown). These data demonstrate that the embryonic lethality of *Gpr126*^Δ*7/*Δ*7*^-null mutants is due to defective placenta formation resulting from loss of Gpr126 function in trophoblast cells.

### *Gpr126*^Δ*7/*Δ*7*^ mutant placentas show maternal vascular abnormalities in the labyrinth and poorly remodeled spiral arteries

To address how Gpr126 contributes to placental development, we first examined the morphology of *Gpr126*^Δ*7/*Δ*7*^ mutant placentas at E11.5, before embryonic death occurs. Histological examination by hematoxylin and eosin (H&E) staining revealed grossly normal organization of the three main placental layers: the decidua basalis, the JZ, and the innermost labyrinth (fig. S13, A and B). High-magnification analysis revealed a similar intricately branched network of fetal and maternal blood spaces in the labyrinth (fig. S13, A′ and B′) but smaller decidual spiral arteries (fig. S13, A″ and B″) in mutant placentas.

**Fig. 6.**
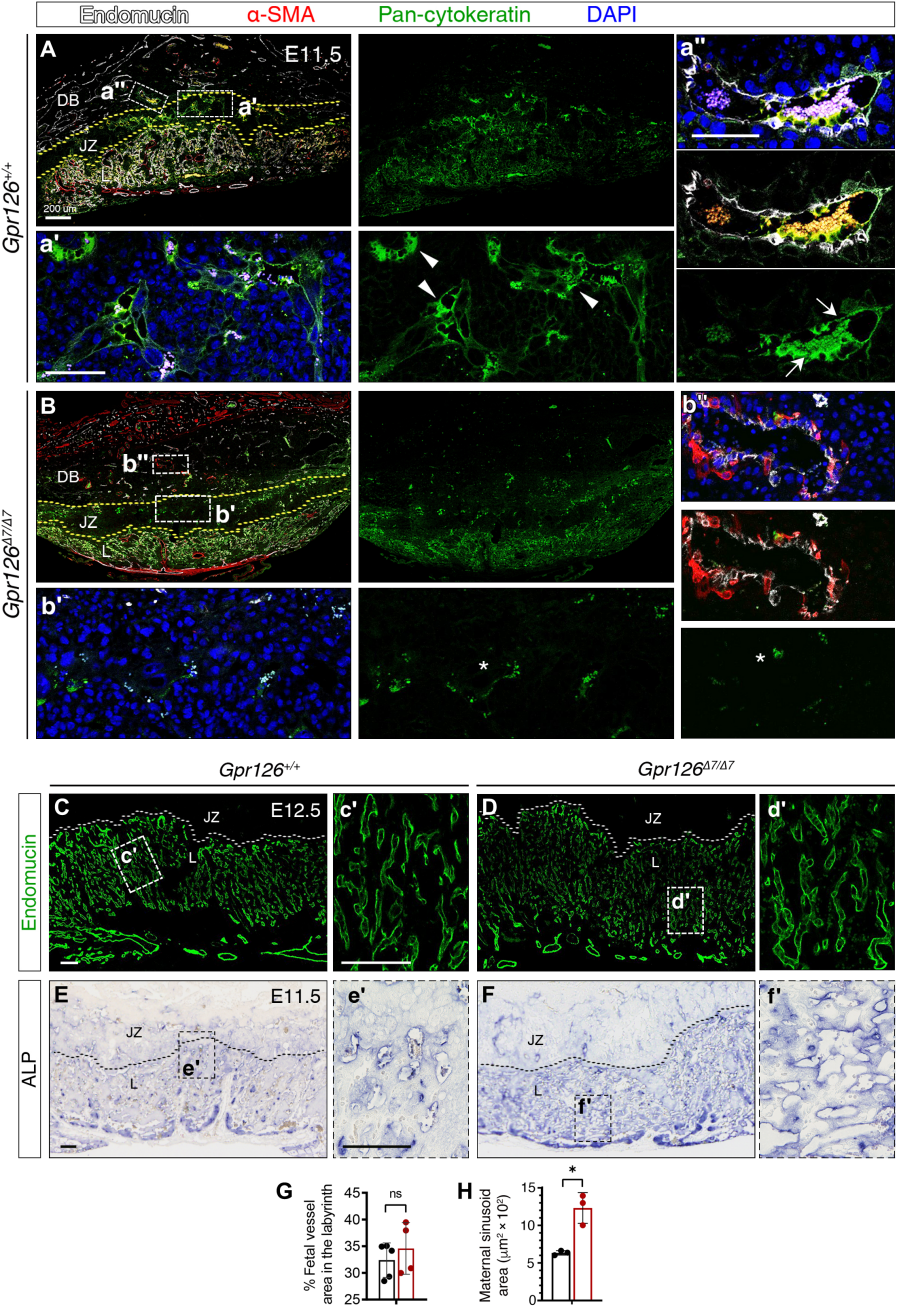
*Gpr126*^Δ*7/*Δ*7*^ mutant placentas show defective maternal spiral arteries remodeling and aberrant organization of maternal sinusoids in the labyrinth. (**A** to **B″**) E11.5 *Gpr126^+/+^* (A) and *Gpr126*^Δ*7/*Δ*7*^ (B) sagittal placental section immunostainings. Endomucin (white) labels maternal endothelial cells in the decidua basalis (DB) and fetal endothelial cells in the labyrinth (L); pan-cytokeratin (green) marks trophoblasts in DB, JZ, and L; α-SMA (red) detects unremodeled spiral arteries in DB and maternal canals in L. Sections were DAPI counterstained (blue). The yellow dotted lines delineate the borders of JZ with DB and L. (A′, A″, B′, and B″) High magnifications corresponding to boxed areas. Arrowheads in (A′) indicate pan-cytokeratin–positive trophoblasts in JZ; arrows in (A″) mark trophoblasts surrounding maternal spiral arteries in the proximal DB; asterisks in (B′ and B″) indicate reduced pan-cytokeratin expression. (**C** to **D′**) E12.5 *Gpr126^+/+^* (C) and *Gpr126*^Δ*7/*Δ*7*^ (D) sagittal placental sections. Endomucin immunostaining (green) in fetal endothelial cells of L. The white dotted lines delineate the JZ and L border. Boxed regions (C and D) are magnified in (′). (**E** to **F′**) ALP staining on E11.5 *Gpr126^+/+^* (E) and *Gpr126*^Δ*7/*Δ*7*^ (F) sagittal placental sections. Maternal blood sinusoids in L are detected by ALP activity of the syncytiotrophoblast layer. Black dotted lines delineate the JZ and L border. Boxed regions (E and F) are magnified in (′). (**G**) Quantification of fetal vessel area in relation to total placental L area in E12.5 *Gpr126^+/+^* and *Gpr126*^Δ*7/*Δ*7*^ placentas. Data are means ± SD (*n* = 5 *Gpr126^+/+^* and 4 *Gpr126*^Δ*7/*Δ*7*^ placentas). Statistical significance: Unpaired Student’s *t* test. (**H**) Quantification of maternal sinusoid area in L of E11.5 *Gpr126^+/+^*and *Gpr126*^Δ*7/*Δ*7*^ placentas: Sinusoids are larger in mutant placentas. Data are means ± SD (*n* = 3 *Gpr126^+/+^* and 3 *Gpr126*^Δ*7/*Δ*7*^ placentas). Statistical significance: unpaired Student’s *t* test (*P < 0.05). Scale bars, 100 μm; otherwise, it is indicated.

Because the remodeling of maternal spiral arteries is critical to establish the definitive uteroplacental circulation (*51*), and key Spa-TGC markers were down-regulated in *Gpr126*^Δ*7/*Δ*7*^ mutant placentas, we then analyze the morphology and architecture of decidual spiral arteries in greater detail. We stained E11.5 WT and *Gpr126*^Δ*7/*Δ*7*^ mutant placentas with antibodies against endomucin to label maternal endothelial cells in the decidua and fetal endothelial cells in the labyrinth, α-smooth muscle actin (α-SMA) to mark unremodeled spiral arteries in the decidua and maternal canals in the labyrinth, and pan-cytokeratin to identify trophoblast cells. We detected strong cytokeratin staining in the JZ (Fig. 6, A and A′) and surrounding endomucin-positive but SMA-negative maternal spiral arteries in the decidua of the WT placenta (Fig. 6, A and A″). In contrast, *Gpr126*^Δ*7/*Δ*7*^ placentas show a severe reduction in trophoblast cells in both the JZ (Fig. 6, B and B′) and around the maternal spiral arteries. Consistent with this, strong endomucin and SMA staining were found in mutant spiral arteries, indicative of impaired remodeling (Fig. 6, B and B″). These results suggest that trophoblast invasion and spiral artery remodeling depend on Gpr126.

The placental labyrinth is the site of nutrient and gas exchange between the maternal and fetal circulatory systems. Defective labyrinth formation is a leading cause of placental failure, fetal growth restriction, and death (*38*, *52*). The crucial role of the labyrinth layer in sustaining embryonic development, together with the detection of *Gpr126* expression in the S-TGCs within this tissue, prompted us to examine the labyrinth organization of *Gpr126*^Δ*7/*Δ*7*^ mutant placentas. Immunostaining for endomucin, which labels fetal endothelial cells, revealed an intricate vascular branching at E12.5 similar to control littermates (Fig. 6, C, D, and G). The size of trophoblasts-lined maternal sinusoids was evaluated by the endogenous ALP activity of the syncytiotrophoblast layer at the maternal-fetal interface (fig. S14G) (*53*). Maternal blood sinusoids were enlarged in mutant placentas (Fig. 6, E, F, and H), which may diminish the surface area available for material exchange between the maternal and fetal blood niches. To visualize the blood pools in the placental labyrinth, we injected the fluorescent dye rhodamine 123 into pregnant females and analyzed the fluorescence intensity derived from dye accumulation in E10.5 placentas, before embryonic death. *Gpr126*^Δ*7/*Δ*7*^ placentas showed increased blood accumulation in the labyrinth, leading to large opaque areas and lower fluorescence intensity (fig. S13, C to F and I). These results indicate that Gpr126 is required for the organization of the trophoblast-lined maternal sinusoid spaces.

To determine whether the exchange of gases between maternal and fetal blood was affected, we measured hypoxia in *Gpr126*^Δ*7/*Δ*7*^ placentas by hypoxyprobe staining. Pimonidazole immunostaining revealed the JZ as the main hypoxic area (*54*), together with the P-TGC layer and the decidua (fig. S14, A to B′). Comparison between control and *Gpr126*^Δ*7/*Δ*7*^ mutant placentas revealed no significant differences in staining intensity or distribution (fig. S14, C and D). We next assessed maternal-fetal nutrient transport by measuring expression of the Glut1 glucose transporter (*55*). At E12.5, Glut1 was ubiquitously expressed in the placenta (*56*, *57*), but protein levels were similar in control and mutants (fig. S14, E to F′). To measure transplacental passage efficiency, we injected pregnant females with the fluorescent dye rhodamine 123 and examined embryo fluorescence after 2 hours. Dye accumulation was similar in E11.5 *Gpr126*^Δ*7/*Δ*7*^ mutants and control siblings (fig. S14, G, H, and J). Similarly, qRT-PCR analysis revealed no effect on key genes expressed in the interhaemal membrane, the interface that mediates selective and bidirectional exchange between the fetal and maternal circulations (fig. S14, G and H) (*52*). These findings indicate that Gpr126 is required for the organization of the trophoblast-lined maternal sinusoid spaces during placental development but does not appear to influence transplacental transport capacity.

## DISCUSSION

aGPCRs form a large family of GPCRs essential for multiple biological processes. Lack of knowledge about the function of specific receptors has hampered their development as therapeutic drug targets. Here, we reveal a previously unknown role for Gpr126 in placental development. Earlier studies reported a requirement for Gpr126 in PNS and heart development in zebrafish and mice (*4*, *5*, *7*, *8*, *15*). However, our results demonstrate that cardiac abnormalities associated with global *Gpr126* inactivation are secondary defects arising from placental insufficiency, highlighting the existence of an essential placenta-heart axis (*58*, *59*). Our analysis of conditional loss- and gain-of-function mutant mice shows that heterozygous and homozygous *Gpr126* mutant embryos associated with *Gpr126*-null placentas die around E13.5, like global knockouts, whereas homozygous mutant embryos with a heterozygous placenta survive to birth. Moreover, restoring Gpr126 function in the placental trophoblast lineage is sufficient to rescue the embryonic lethality of homozygous mutants. We provide evidence for roles of Gpr126 in the organization of the maternal blood sinusoids in the labyrinth and on vascular remodeling in the decidua. The dispensability of Gpr126 for heart development is supported by our findings in zebrafish, showing that larvae homozygous for *gpr126* loss-of-function alleles have no trabeculation defects and survive to adulthood. We also show that the adhesive CUB and PTX domains within the NTF are dispensable for placentation but critical for PNS myelination.

In previous mouse studies, *Gpr126* exons 3 and 4 (2 and 3 in the original publication) were replaced with a 4.6-Kb β-geo-puromycin cassette, thus generating a null allele; 8% of homozygous carriers survived to birth, showing abnormal limb joint contractures and uncoordinated locomotion due to defective PNS myelination, and died postnatally (*15*). Our *Gpr126*^Δ*3*,*4/*Δ*3*,*4*^ mutants survive embryogenesis and recapitulate these limb joint contractures at birth. The *Gpr126*^Δ*3*,*4*^ allele encodes an mRNA that is likely translated into a shorter protein lacking the CUB and PTX domains. The survival of *Gpr126*^Δ*3*,*4/*Δ*3*,*4*^ mutants reveals a distinct Gpr126 domain dependence in the embryo proper and the placenta, with the Gpr126 CUB and PTX domains being essential for PNS myelination but dispensable for embryonic progression. The joint phenotype of *Gpr126*^Δ*3*,*4/*Δ*3*,*4*^ and *Gpr126^fl/fl^;Sox2^Cre/+^* mutants is reminiscent of the human disease AMC, defined by the presence of congenital joint contractures in two or more body regions and sometimes associated with fetal or neonatal death (*13*). Loss-of-function mutations in human *GPR126* have been identified in three consanguineous families affected by lethal AMC, providing the first evidence that *GPR126* mutations disrupt myelination in humans (*13*). AMC is thought to be related to decreased fetal movement in the uterus. It is conceivable that abnormal PNS development caused by *GPR126* abrogation could diminish fetal movement, resulting in congenital contractures. The *Gpr126*^Δ*3*,*4*^ mouse line could therefore be used to model AMC pathogenesis in humans. In this regard, although AMC patients have a normal life span (*60*), all fetuses and newborns carrying homozygous *GPR126* mutations were aborted or dead at birth, concurrent with intrauterine growth retardation or severe preeclampsia (*13*). Thus, the requirement for GPR126 in the placenta might be common to mice and humans, with its loss here causing fetal death, whereas disruption of GPR126 function in embryonic PNS development could lead to AMC. Confirmation of this hypothesis would open an avenue for future research and a future clinical application in preimplantation genetic diagnosis.

Despite the large number of *gpr126* mutant lines generated in zebrafish, no cardiac defects have been reported (*2*, *3*, *4*, *8*, *31*, *32*). The evidence for a role of gpr126 in zebrafish heart development is based on morpholino experiments, suggesting that the NTF signals to control trabeculation (*5*). Our *gpr126^bns341^* allele lacks exons 7 and 8, and a shorter *gpr126* mRNA was detected in mutants, suggesting the possible existence of a protein containing only the signal peptide, CUB, and PTX domains. Homozygous *gpr126^bns341^* larvae have ear defects but normal heart development, a phenotype resembling that of *gpr126^st86^* mutants that harbor a PTC codon in exon 7 (*3*). Likewise, we found no heart phenotype in homozygous *gpr126^bns342^* mutants, which lack the promoter and fail to transcribe *gpr126*. Recently, a mechanism termed transcriptional adaptation has been described in zebrafish, by which aberrant mRNA degradation triggers the up-regulation of a potential compensatory gene or genes able to provide normal function (*34*). However, neither the *gpr126^bns341^* allele, expressing a shorter mRNA, nor the “promoter-less” *gpr126^bns342^* allele, with no mRNA expression, would promote the up-regulation of potential compensatory genes. In either case, our data indicate that *gpr126* inactivation allows zebrafish development and survival.

Previous studies with *Gpr126* knockout mouse lines have shown that homozygous deletion leads to embryonic death associated with cardiac abnormalities. One study described thinning of the ventricular wall at E11.5 and suggested that ventricular function might fail under increased load (*7*). Another study reported ventricular wall and trabeculae thinning and aberrant mitochondrial function in *Gpr126*-deficient hearts and suggested that the endocardium communicates through Gpr126 to regulate metabolism, contractility, and trabeculation (*5*). However, there are no previous reports on the tissue-specific requirement for *Gpr126*. Our global and conditional knockout models both efficiently blocked *Gpr126* mRNA expression. The global *Gpr126*^Δ*7/*Δ*7*^ mutants (lacking exon 7 and with a PTC in exon 8) had thinner ventricular walls and trabeculae and were dead by E13.5; nevertheless, chamber patterning, cardiac metabolism, and cellular proliferation were unaffected. In the conditional *Gpr126^flox^* model (in which exon 7 is flanked by loxP sites), endocardium-specific *Gpr126* inactivation (*Gpr126*^*flox/*Δ*7*^ or *Gpr126^flox/flox^*) in the *Nfatc1^pan-Cre^* or the *Tie2^Cre^* lines did not recapitulate the cardiac defects observed in the global *Gpr126*^Δ*7/*Δ*7*^ mutants. In addition, *Gpr126* ablation in the early cardiogenic mesoderm with the *Mesp1^Cre^* driver did not disrupt development or viability, indicating that cardiac Gpr126 is not essential for embryogenesis. Last, death of *Gpr126*-null embryos could not be rescued by endothelium-specific *GPR126* expression, consistent with the notion that lethality is not due to defective development of the heart. Moreover, the normal ECG, echocardiography, and histology in *Gpr126^fl/fl^;Tie2^Cre^* hearts exclude a requirement for *Gpr126* in adult heart homeostasis. Together, these findings argue against a primary requirement of Gpr126 for heart development or function.

Our data support an extraembryonic requirement for Gpr126. First, *Gpr126* is expressed in the SpT and all TGC subtypes of the mouse placenta. Second, epiblast-specific deletion of *Gpr126* by paternally inherited *Sox2^Cre^* allows survival of homozygous mutant offspring (*Gpr126*^*flox/*Δ*7*^;*Sox2^Cre/+^*) with a *Gpr126* heterozygous placenta. In contrast, germline deletion of *Gpr126* via maternal inheritance of *Sox2^Cre^* leads to fully penetrant embryonic lethality. The idea that placental trophoblast defects cause death in *Gpr126*^Δ*7/*Δ*7*^-null embryos is also supported by the failure of GPR126 function restoration in the epiblast to rescue embryonic lethality; *GPR126*-expressing embryos associated with a *Gpr126*-deficient placenta are dead by E13.5, resembling global knockouts.

In the hemochorial placentas of humans and rodents, fetal vessels are lined by endothelial cells, whereas maternal blood spaces are built by invasive fetal trophoblast cells (*42*). *Gpr126*^Δ*7/*Δ*7*^ mutant placentas had abnormally enlarged maternal sinusoids, resulting in accumulation of maternal blood in the labyrinth. Moreover, they showed reduced expression of proteases involved in trophoblast invasion and maternal spiral artery remodeling (*Mmp9*, *Cts7*, and *Cts8*), concomitant with fewer trophoblasts in the JZ and around maternal spiral arteries. GlyTs were also abnormally distributed around decidual spiral arteries. Defective trophoblast invasion and spiral artery remodeling are key features of preeclampsia, and a recent study reported altered *GPR126* expression in preeclamptic human placentas (*61*). Preeclampsia manifests clinically as gestational hypertension, proteinuria, and vascular dysfunction. Given the relatively early embryonic lethality, *Gpr126*^Δ*7/*Δ*7*^ mutants likely die as a result of a severe disruption of the maternal vascular remodeling before clinical manifestations arise in the mother.

**Fig. 7.**
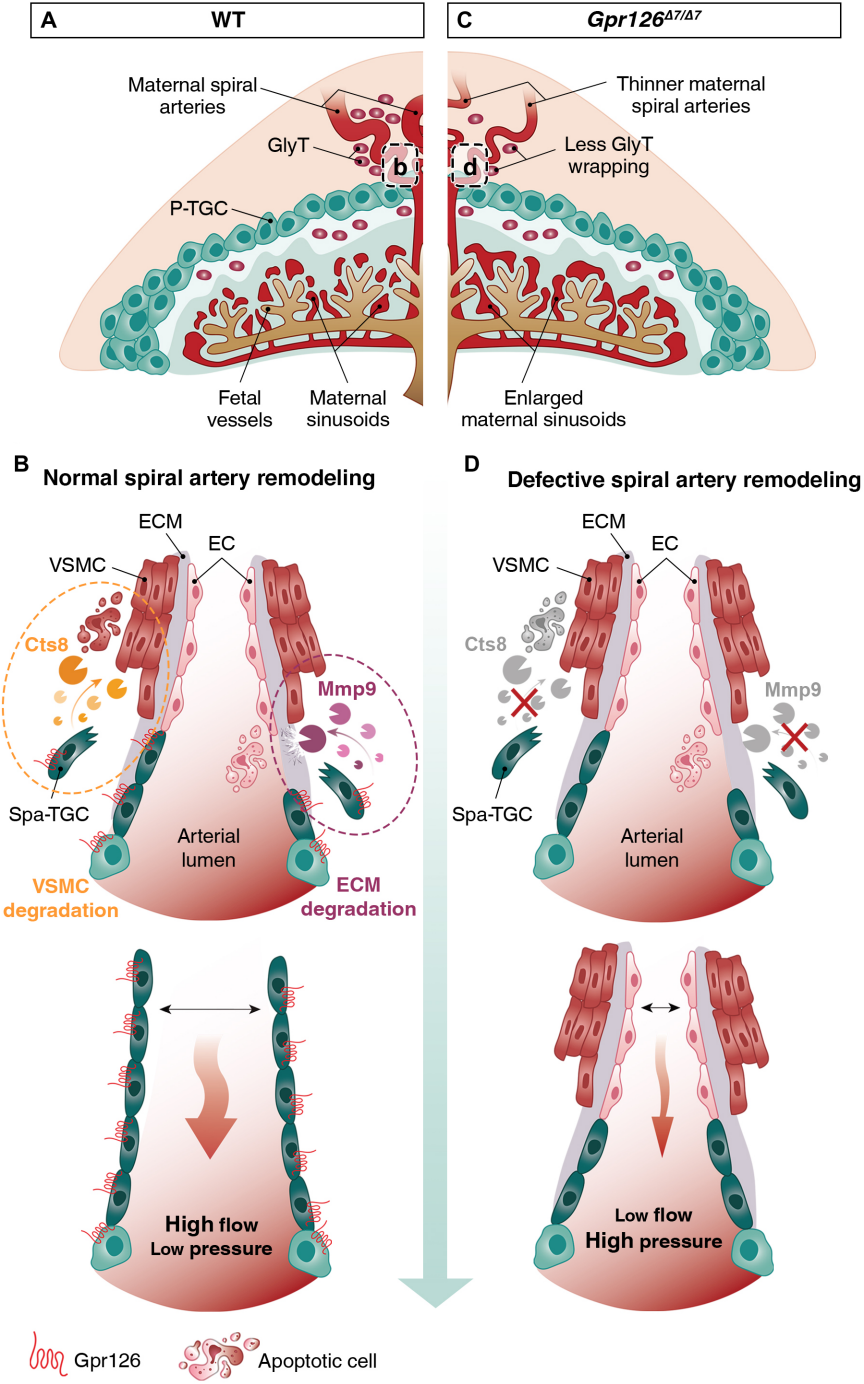
Model of Gpr126 function in the remodeling of the maternal vasculature of the mouse placenta. (**A** to **D**) Illustrations of an E12.5 WT (A) and a *Gpr126*^Δ*7/*Δ*7*^ (C) placenta. In a WT placenta (A and B), *Gpr126* endows TGCs with invasive behavior by regulating the production of *Mmp9*, together with the capacity to degrade vascular smooth muscle cells through the expression of *Cts8*. Both activities are required for uterine trophoblast invasion and vascular remodeling during placental development, through which Spa-TGCs replace the maternal endothelial lining and GlyTs congregate around spiral arteries, transforming the maternal spiral arteries from narrow high-resistance, low-flow vessels into dilated vessels, allowing higher blood flow at lower pressure. The *Gpr126*-null placentas (C and D) fail to express *Mmp9* and *Cts8*, thus impairing trophoblast migration and spiral artery adaptation. Fewer GlyTs wrapped around the unremodeled maternal spiral arteries. As a result of the altered uteroplacental hemodynamics, increased blood pressure triggers an enlargement of the maternal sinusoids. EC, endothelial cell; ECM, extracellular matrix; GlyT, glycogen trophoblasts; VSMC, vascular smooth muscle cell.

We propose that Gpr126 regulates the transcription of proteases involved in trophoblast invasion of the maternal decidua and maternal spiral artery remodeling during placental development (Fig. 7A). Mmp9 participates in the breakdown of collagen IV, the main component of the maternal basal membrane, and plays a critical role in decidual ECM remodeling and cell migration (*62*) (Fig. 7B). Cts8 mediates the loss of SMA in the vessel wall, thus facilitating the transformation of narrow high-resistance, low-flow vessels into dilated low-resistance, high-flow vessels that lack maternal vasomotor control (Fig. 7B). Through the secretion of various proteases, Spa-TGCs lastly replace the endothelial lining of maternal spiral arteries and acquire a pseudoendothelial phenotype (Fig. 7B) (*63*, *64*), resulting in increased blood flow to the developing embryo (*65*). A proportion of GlyT cells also invade the decidua from the JZ and congregate around the maternal spiral arteries (Fig. 7A) (*66*). *Gpr126* inactivation reduces the expression of *Mmp9* and *Cts8*, leading to weak trophoblast invasion, defective vascular remodeling, and impaired GlyT cell accumulation around the maternal spiral arteries (Fig. 7, C and D). Narrow high-resistance, nonremodeled vessels associate with high-perfusion blood pressure into the labyrinth and shear stress in the trophoblast layer, likely resulting in an enlargement of maternal blood spaces and aberrant labyrinth architecture (Fig. 7D). These defects compromise embryonic progression and might be involved in the onset of pregnancy diseases like preeclampsia. Our findings highlight the known connection between the placenta and the heart, referred to as the “placenta-heart axis” and based on the interdependence of the development of both organs and their requirement for common signaling pathways (*67*, *68*). The enlargement of the heart chambers and thinning of the ventricular walls observed in *Gpr126*^Δ*7/*Δ*7*^ embryos have been also described in other global knockout mice as secondary phenotypes linked to a primary placental requirement (*52*, *69*, *70*). Transcriptome profiling of *Gpr126*^Δ*7/*Δ*7*^ mutant ventricles revealed an enrichment in DEGs involved in angiogenesis and vasculature development. The endocardium senses and responds to blood flow during cardiac development and homeostasis (*71*). Hence, we suggest that transcriptional alteration of endocardial gene expression in *Gpr126*^Δ*7/*Δ*7*^ mice could be a mechanosensitive transcriptional response to altered placental hemodynamics in mutant placentas. Deficient remodeling of uterine spiral arteries can lead to increased resistance of placental vasculature and cardiac overload, thus increasing the mechanical force acting on the heart (*58*). aGPCRs are of ancient origin and are found across vertebrates (*72*). Their signaling versatility is reflected in the observation that although mammalian and fish Gpr126 have a conserved role in PNS myelination (*8*, *15*), mammalian Gpr126 has acquired a new function in mammals, related to spiral artery remodeling during placental development.

## METHODS

### Mice

Animal studies were approved by the Centro Nacional de Investigaciones Cardiovasculares (CNIC) Animal Experimentation Ethics Committee and by the Community of Madrid (ref. PROEX 118/15). All animal procedures conformed to EU Directive 2010/63EU and Recommendation 2007/526/EC regarding the protection of animals used for experimental and other scientific purposes, enacted in Spanish law under Real Decreto 1201/2005. Mouse strains used in this study are as follows: *Gpr126*^Δ*3*,*4*^, *Gpr126*^Δ*7*^, *Gpr126^flox^*, *GPR126^GOF^*, *Tie2^Cre^* (*24*), *Nfatc1^pan-Cre^* (*23*), *Nkx2.5^Cre^* (*29*), *Mesp1^Cre^* (*25*), and *Sox2^Cre^* (*48*). The *Gpr126*^Δ*3*,*4*^, *Gpr126*^Δ*7*^, and *Gpr126^flox^* mouse lines were generated with CRISPR-Cas9 technology. *GPR126^GOF^* mice were generated by homologous recombination in mouse embryonic stem (mES) cells. cDNA encoding human *GPR126* was provided by Torsten Schöneberg’s laboratory (*9*). *GPR126* was cloned into a modified version of the *pROSA26-1* plasmid, preceded by a loxP-flanked *PGK-Neo^R^-STOP* cassette, and followed by an *IRES-EGFP* cassette (fig. S6A). Gene targeting, performed in G4 mES cells, was confirmed by Southern blotting with external 5′- and 3′-hybridization probes (fig. S6B). Mice were generated by injecting targeted cells into B6CRL blastocysts to generate chimeras that were then analyzed for germline transmission. The selected animals were backcrossed to the C57BL/6 background.

### Generation of *Gpr126* mouse mutants

CRISPR RNA (crRNA) sequences were designed with the CRISPOR-TEFOR online tool (http://crispor.tefor.net/crispor.py). The annealed two-part synthetic crRNA [Alt-R^R^ CRISPR-Cas9 crRNA, 2 nmol, Integrated DNA Technologies (IDT)] and tracrRNA (Alt-R^R^ CRISPR-Cas9 tracrRNA, 5 nmol, IDT, 1072532) were diluted in microinjection buffer [1 mM tris HCl (pH 7.5), 0.1 mM EDTA] and incubated with *Sp* Cas9 nuclease (IDT, 1081058). To generate the *Gpr126^flox^* line, two complementary and asymmetric single-stranded oligodeoxynucleotides (ssODNs) were designed according to the published guidelines (*73*) as custom synthetic genes (Megamer single-stranded Gene Fragments, IDT). The final component concentrations were crRNA and tracrRNA (0.61 pmol/μl), Cas9 protein (30 ng/μl), and ssODN (10 ng/μl). Sequences of the crRNAs and ssODNs used in this study and their genomic binding sites are listed in table S5 (sheet 1). Microinjections were performed at one cell–stage fertilized C57BL/6 mouse embryos (*74*). To generate the *Gpr126^flox^* line, microinjected zygotes were treated with RS-1 (7.5 μM) (*75*). Pups were screened for the targeted mutation or insertion by PCR analysis and sequencing, and the selected founders were backcrossed to the C57BL/6 background.

### Zebrafish

All experiments with zebrafish (*Danio rerio*, strain Tüb/AB) were performed in accordance with German and Spanish Animal Protection Laws approved by the regional governments’ animal protection committees. Zebrafish were raised and maintained under standard conditions. All experiments were performed on zebrafish embryos or larvae between 48 hpf and 5 dpf. Transgenic lines used in this study were *Tg(kdrl:Hsa.HRAS-mCherry)* (*35*) and *Tg(myl7:EGFP-Hsa.HRAS)* (*36*). The mutant zebrafish lines *gpr126^bns341^* and *gpr126^bns342^* were generated with CRISPR-Cas9 technology.

### Generation of *gpr126* zebrafish mutants

*gpr126* zebrafish mutants were generated using the CRISPR-Cas9 system. crRNA sequences were designed with the CRISPOR-TEFOR online tool, and those with higher specificity and efficiency scores were selected. To generate the *gpr126^bns341^* (*gpr126*^Δ*7*,*8*^) zebrafish line, one sense and one antisense oligonucleotide (10 μM) were annealed (5 min at 95°C and overnight at room temperature) and cloned into a Bsm BI [New England Biolabs (NEB), R0739]–digested pT7–guide RNA (gRNA) vector (Addgene, plasmid 46759) using T4 ligase (Promega, M1801) for 2 hours at room temperature. The gDNA template was linearized using Bam HI-HF (NEB, R3136S), purified using a Gel DNA Recovery kit (Zymo Research, D4001), and transcribed using the MEGAshortscript T7 Transcription Kit (Invitrogen, AMB13345) following the manufacturer’s instructions for 5 hours at 37°C. To generate the *gpr126^bns342^* (*gpr126^promoterless^*) zebrafish line, double gRNAs were designed to flank the region to be deleted (*76*). *gpr126*-specific oligonucleotides containing the T7 promoter sequence (5′-TAATACGACTCACTATA-3′), the 20-base target site without the protospacer adjacent motif (PAM) sequence, and a complementary region, were annealed to a constant oligonucleotide encoding the reverse complement of the tracrRNA tail (5 min at 95°C, slow cooling at room temperature, and 20 min at 4°C). The single-stranded DNA overhangs were filled in with T4 DNA polymerase (NEB, M0203), bovine serum albumin (BSA; NEB), and deoxynucleotide triphosphates (dNTPs) (10 μM) (Promega, U1511) for 20 min at 12°C. The resulting gRNA template was purified using a QIAquick nucleotide removal kit (QIAGEN, 28304), and in vitro transcription was performed as previously described. Sequences of the gRNAs and their genomic binding sites are listed in table S5 (sheet 1). Cas9 mRNA was transcribed from linearized pT3TS-nCas9n template (Addgene, plasmid 46757) using the mMESSAGE mMACHINE T3 kit (Invitrogen, AM1348) for 3 hours at 37°C according to the manufacturer’s instructions. The microinjection solution for the generation of mutant lines contained each gRNA (75 ng/μl; 2 for the *gpr126^bns341^* line and 4 for the *gpr126^bns342^* line), Cas9 mRNA (200 ng/μl), and phenol red in a final volume of 10 μl. The microinjection solution was injected into the yolk of zebrafish AB zygotes at the one-cell stage using a Narishige IM 300 microinjector. The injected embryos were genotyped at 24 hpf to examine the presence of deletions in the targeted regions. If the deletion was detected, then the embryos were grown to adults for founder identification.

### Genotyping

Mouse genomic DNA was extracted from tail-tip biopsies of weaned or adult mice or from the yolk sac or tail tip of embryonic or fetal samples. Zebrafish genomic DNA was extracted from clipped adult fins or from whole embryos. The primer sequences used for genotyping all mouse and zebrafish lines are listed in table S5 (sheet 2). To screen for mouse and zebrafish carriers, PCR products were sent for sequencing to the Eurofins LightRun Sequencing Service.

### Tissue processing, histology, and ISH

Mouse embryos, hearts, and placentas were fixed overnight at 4°C in 4% paraformaldehyde (PFA) (Electron Microscopy Sciences, 50980487). Adult hearts were perfused with heparin [5 U/ml in phosphate-buffered saline (PBS)] and fixed in 4% PFA for 48 hours at room temperature. All samples were dehydrated through a series of ethanol solutions of increasing concentrations and embedded in paraffin following standard protocols. Serial sections (7 μm) were distributed on several slides, such that each slide contained sections of several levels of the corresponding organ. Embryos used for WISH were washed in PBS containing 0.1% Tween 20 and dehydrated by washing in a methanol series of increasing concentrations. H&E, Masson’s trichrome, and periodic acid–Schiff staining were performed using standard procedures at the CNIC Histology Facility. ISH on paraffin sections and WISH were performed as described previously (*77*, *78*). Antisense RNA probes were designed to be exon spanning or complementary to the 5′UTR or 3′UTR sequences. Digoxigenin-labeled RNA probes were transcribed by in vitro transcription of T7 or Sp6 polymerase using the DIG RNA labeling mix (Roche, 11277073910) following the manufacturer’s instructions and purified using the Illustra AutoSeq G-50 Kit (Cytiva, 27534001). Primer sequences used for probe generation are listed in table S5 (sheet 3).

### Immunofluorescence

Paraffin sections (7 μm) were rehydrated. For antigen retrieval, sections were boiled for 15 min in 10 mM citrate buffer (Sigma-Aldrich, 25114; pH 6). If needed, endogenous peroxidases were quenched with 1% hydrogen peroxide solution (Sigma-Aldrich, H1009) in methanol for 40 min. Tissues were permeabilized with 0.3% Triton X-100 (Bio-Rad, 1610407) in PBS before blocking with 3% BSA (Sigma-Aldrich, A9418), 5% goat serum (Vector Laboratories, S-1000), 20 mM MgCl_2_, and 0.3% Tween 20 in PBS for 1 hour at room temperature. Sections were incubated with primary antibodies overnight at 4°C, followed by 1 hour of incubation at room temperature with a fluorescent dye–conjugated secondary antibody. 5-Bromo-2′-deoxyuridine (BrdU) and pimonidazole stainings were performed using Tyramide Signal Amplification Plus Fluorescein (PerkinElmer, NEL741B001KT) for 3 min at room temperature according to the manufacturer’s instructions. Samples were incubated with Alexa Fluor 647–conjugated Isolectin GS-IB4 from *Griffonia simplicifolia* (Thermo Fisher Scientific, I32450) and rhodamine-conjugated wheat germ agglutinin (WGA) (Vector Laboratories, RL-1022) in PBS (1:200) for 2 hours at room temperature before 4′,6-diamidino-2-phenylindole (DAPI) counterstaining. Slides were mounted in Fluoromount (Southern Biotech, 0100-01) for imaging. All antibodies used are listed in table S5 (sheet 4).

### Quantitative reverse transcription PCR (qRT-PCR)

Mouse embryos, hearts, and placentas were dissected in ice-cold PBS, mixed with TRI Reagent Solution (Invitrogen, AM9738) containing steel beads, and homogenized for 5 min at 50 Hz using a TissueLyser LT Adapter (QIAGEN, 85600). Total RNA was purified using the direct-zol RNA MiniPrep kit (Zymo Research, R2051). cDNA was obtained from 1 μg of total RNA using the High Capacity cDNA Reverse Transcription Kit (Applied Biosystems, 4368814). Zebrafish embryos were homogenized in TRI Reagent Solution using a NextAdvance Bullet Blender Homogenizer (Scientific Instrument Services), and RNA was isolated by phenol-chloroform extraction. cDNA was obtained from 600 ng of total RNA using the Maxima First-Strand cDNA synthesis kit (Thermo Fisher Scientific, K1641). Two to five biological replicates and three technical replicates were performed for mouse gene expression analysis. One to three biological replicates and three technical replicates were performed for zebrafish gene expression analysis. The primer sequences used for qRT-PCR are listed in table S1 (sheet 5). Real-time PCRs were performed using Power SYBR Green PCR Master Mix (2×) (Applied Biosystems, 4367659) in a total reaction volume of 10 μl. For mouse samples, qPCR was performed at the CNIC using an ABI PRISM 7900HT FAST Real-Time PCR System (Applied Biosystems). For zebrafish samples, qPCR was performed using a CFX Connect Real-Time System (Bio-Rad). Relative expression levels were calculated using comparative Ct values after normalizing to the geometric mean of internal control genes (*Gapdh* or β*-actin* for mouse samples and *rpl13a* for zebrafish). Relative gene expression data were analyzed using the 2^−ΔΔCT^ method (*79*). Statistical comparisons were made by two-tailed unpaired Student’s *t* test in GraphPad Prism version 8, and differences were considered statistically significant at *P* < 0.05. Data are presented as means ± SD.

### RNA-seq analysis

E12.5 ventricles were collected from embryonic hearts after removal of the atria and the valve region in sterile ice-cold PBS. Four pools of *Gpr126^+/+^* and *Gpr126*^Δ*7/*Δ*7*^ embryonic ventricles (four ventricles per pool) were placed in TRI Reagent Solution containing steel beads and homogenized with a TissueLyser LT Adapter for 5 min at 50 Hz. RNA was extracted with the PicoPure RNA Isolation kit (Applied Biosystems, KIT0204). RNA integrity was verified with an Agilent 2100 Bioanalyzer instrument (Agilent Technologies). cDNA libraries were prepared using the NEBNext Ultra RNA Library preparation kit (NEB). Libraries were sequenced on a HiSeq 2500 system (Illumina) to generate 60-base single reads. Next-generation sequencing (NGS) experiments were performed at the CNIC Genomics Unit. Sequenced reads were quality control filtered and preprocessed using Cutadapt v1.6 (*80*) to eliminate adaptor remains. The resulting reads were mapped against the mouse transcriptome (GRCm38 assembly, Ensembl release 70) and quantified using RSEM (RNA-Seq by Expectation Maximization) v1.2.3 (*81*). Differential gene expression was analyzed using the Bioconductor EdgeR package (*82*). One global knockout sample was classified as an outlier on the basis of multidimensional scaling plots produced during the pipeline output and was discarded. Only genes expressed at a minimal level of 1 count per million in at least two samples were considered for differential expression analysis. Changes in gene expression were considered significant if associated with a Benjamini-Hochberg adjusted *P* value <0.05 (table S2). NGS data analysis was performed by the CNIC Bioinformatics Unit. For the set of DEGs, GO, and Kyoto Encyclopedia of Genes and Genomes, pathway enrichment analyses were performed in GO-Elite (www.genmapp.org/go_elite/; 1.2.5, EnsMart77Plus database version) (*83*) with a permuted *P* value cutoff <0.05 (table S3). Bar charts representing the *z* score values of the terms belonging to the “biological processes” category were generated with GraphPad Prism version 8.

### Accession number

Data are deposited in the National Center for Biotechnology Information Gene Expression Omnibus database under accession number GSE166903.

### Sequence similarity analysis of mouse and zebrafish *gpr126* loci

Sequence similarity was analyzed using the Bioinformatics and Deep Sequencing Platform in the MPI-HLR, as described in (*34*). To identify similarity to query nucleotide sequences of zebrafish *gpr126* (ENSDARG00000054137) (table S4, sheet 1) and mouse *Gpr126* (ENSMUSG00000039116) (table S4, sheet 2), the longest respective transcripts were selected (ENSDART00000145927 and ENSMUST00000041168.5, respectively) and compared with the whole genome using BLASTn (*84*). Genes were considered similar to the query gene when a partial match was identified inside the target gene body or the promoter region (2 kb upstream of the transcription start site). Several alignment parameters were surveyed to identify the optimal degree of similarity: alignment length, bit score (the combination of alignment quality and length), and *E* value (the probability that the match resulted by chance, when considering the whole target database). Thresholds were established as follows: from 21 onward for alignment length, from 40 to 100 for the bit score, and from 5 × 10^−15^ to 5.5 for the *E* value. These thresholds translate into local nucleotide sequence alignments ranging from 21 to 273 nucleotides in length and 71.06 to 100% identity.

### BrdU, pimonidazole, and rhodamine 123 injections

Pregnant *Gpr126*^Δ*7/+*^ females were intraperitoneally injected with 300 μl of BrdU (BD Pharmigen, 550891) at 10 mg/ml, 2 hours before sacrifice. Rhodamine 123 (Sigma-Aldrich, R8004) was injected subcutaneously (1 μg/g of body weight) into pregnant mice 20 min before sacrifice. The Hypoxyprobe-1 Plus Kit (Chemicon, HP2-100) was used according to the manufacturer’s instructions. The hypoxia marker pimonidazole hydrochloride (PIMO) was injected intraperitoneally (60 mg/kg of body weight) into pregnant mice 30 min before sacrifice.

### Zebrafish embryo preparation for live imaging

Zebrafish embryos were treated from 28 hpf onward with phenylthiourea (Sigma-Aldrich, P7629) to avoid pigmentation and dechorionated just before live imaging using pronase (30 mg/ml) (Sigma-Aldrich, P6911).

### Microscopy and confocal imaging

Whole-mount images were acquired with a Leica MZFL III binocular microscope coupled to an Olympus DP 71 CCD camera and Olympus cellSense software. Bright-field images were obtained with an Olympus BX51 Microscope and Olympus cellSense software. Confocal images were acquired with a Nikon A1R confocal microscope. For in vivo confocal imaging, zebrafish embryos and larvae were mounted and anaesthetized in 1% low-melting agarose (Sigma-Aldrich, A9414) containing 0.2% (w/v) tricaine on glass-bottom dishes. *Z*-plane images were obtained with a spinning disc confocal microscope (Zeiss, CSU-X1 Yokogawa) fitted with a 40× [1.1 numerical aperture (NA)] water immersion objective. The optical section thickness was 1 μm. Three nonconsecutive single-plane images per larvae were taken using a Zeiss LSM780 confocal microscope and a 40× (1.1 NA) water immersion objective. Confocal data were processed with ZEN 2012 software (black edition), and images were analyzed with ImageJ.

### Quantification of cardiac cell proliferation

Cell proliferation in mouse embryonic hearts was assessed by BrdU incorporation. BrdU-positive cells were detected by immunofluorescence following the protocol described in (*85*). ImageJ was used to count total cells and BrdU-positive cells in the compact myocardium, trabecular myocardium (counterstained with α-SMA), and endocardium [counterstained with ETS-related gene (ERG)] in six nonconsecutive sections (three from the right ventricle and three from the left ventricle) from five embryos of each genotype using the ImageJ software. Statistical comparisons were by unpaired two-tailed Student’s *t* test, and differences were considered statistically significant at *P* < 0.05. Data are presented as means ± SD.

### Quantification of hypoxia

Confocal images were binarized by splitting color channels, and mean PIMO intensity was calculated with ImageJ. PIMO-positive areas were selected using the “Otsu dark” intensity threshold (578, 4095), total placental area was selected manually using DAPI staining, and the ratio between them was calculated. Two nonconsecutive sections were measured per placenta. Statistical comparisons were by unpaired two-tailed Student’s *t* test, and differences were considered statistically significant at *P* < 0.05. Data are presented as means ± SD.

### Quantification of the zebrafish ear area

Ear cross-sectional area was calculated from the perimeter drawn with ImageJ. Statistical comparisons were by unpaired two-tailed Student’s *t* test in GraphPad Prism version 8, and differences were considered statistically significant at *P* < 0.05. Data are presented as means ± SD.

### Quantification of maternal and fetal blood space surface areas

To quantify maternal and fetal blood spaces, confocal images were binarized by splitting color channels with ImageJ and selecting the endomucin-containing channel (fetal vessel area) or the WGA-containing channel (maternal sinusoid area). The contrast was enhanced (14% saturated pixels for endomucin and 100% for WGA), and the Gaussian Blur filter was applied (Sigma 1.5 for endomucin; 2 for WGA). The placental labyrinth was selected manually and thresholded (“RenyiEntropy” 524, end), and the total area was measured. The endomucin-positive area was digitally identified and measured by selecting the RenyiEntropy threshold (0, 14,000). The largest black spaces in the labyrinth (corresponding to maternal sinusoids) were digitally identified and measured by selecting the Otsu threshold (0, 12,000). The percentage of fetal vessels (endomucin positive) in the labyrinth was obtained by calculating the ratio of the endomucin-positive area to the labyrinth area. The percentage of maternal sinusoids (dark lacunae) in the labyrinth was obtained by calculating the ratio of the lacunae area to the labyrinth area. Two nonconsecutive sections were measured per placenta. Statistical comparisons were by unpaired two-tailed Student’s *t* test in GraphPad Prism version 8, and differences were considered statistically significant at *P* < 0.05. Data are presented as means ± SD.

### ALP activity and quantification

For comparison of the maternal sinusoid size within the labyrinthine layer, placental sections processed for ALP activity were analyzed. Placentas were dissected on E11.5, fixed in 4% PFA (in PBS), paraffin embedded, and serially sectioned. Dewaxed sections were incubated with BM-Purple substrate (Merck) for 30 min at room temperature in the dark according to (*86*). The cross-sectional area of the maternal sinusoids was measured with Fiji, delineating the sinusoid with a freehand tool and automatically quantified.

### Quantification of rhodamine 123 fluorescence intensity

Rhodamine 123 fluorescence intensity was quantified with ImageJ. Epifluorescence images were converted to 8-bit grayscale, the sample was manually delineated, and mean intensity was calculated. Statistical comparisons were by unpaired two-tailed Student’s *t* test in GraphPad Prism version 8, and differences were considered statistically significant at *P* < 0.05. Data are presented as means ± SD.

### Electrocardiography

ECGs were recorded in anaesthetized mice as previously described (*87*). Briefly, mice were placed supine on a temperature-controlled surface and were anaesthetized with 1.5 to 2% isoflurane. Four-needle electrodes were inserted subcutaneously in the limbs. ECGs were acquired with a MP36R Biopac Systems and analyzed offline with LabChart7 software (AD Instruments, Australia). A high-pass filter setting of 0.5 Hz was applied to remove baseline wander with a bidirectional filtering strategy. The PR interval was measured from the beginning of the positive deflection of the P wave to the peak of the R wave; the QRS interval was measured from the start of the Q wave to the point where the S wave crosses the isoelectric line; and the QTc interval was measured from the start of the Q wave to the point where the T wave reaches 90% of the decline, including correction for RR intervals according to Bazett’s formula for mouse ECGs (*88*). All intervals were calculated as the mean of ~450 ECG measurements. The following formulae were used to calculate short-term QT variability markers such as QT variance (QTvar), the SD of the QT intervals (SDqt), short-term variability of the QT intervals (STVqt), QT variance normalized for mean QT interval (QTVN), QT variability index (QTVI_HR_), and the root mean square of the successive QT interval differences (RMSSDqt)

**Table:** 

**Qtvar** $\frac{1}{n-1}\sum_{i=1}^{n}{\left(QT_{i}-\text{QTm}\right)}^{2}$	**SDqt** $\sqrt{\text{QTvar}}$	**STVqt** $\sum_{i=1}^{n}\frac{|QT_{i+1}-QT_{i}|}{n\sqrt{2}}$
**QTVN** $\frac{\text{QTvar}}{QT_{m}^{2}}$	**QTVI**_**HR**_ log10[(QTvar∕QTm2)(HRvar)HRm2¯]	**RMSSDqt** $\sqrt{\frac{1}{n-1}\sum_{i=1}^{n-1}{\left(QT_{i+1}-QT_{i}\right)}^{2}}$

Results are presented as the means ± SD for 31- to 36-week-old *Gpr126^fl/fl^;Tie2^+/+^* mice (four males and three females) and *Gpr126^fl/fl^;Tie2^Cre/+^* mice (five males and nine females). Statistical significance was determined by unpaired Student’s *t* test subjected to Welch’s correction. To allow for repeated sample assessments, data were analyzed with multilevel mixed-effects models. Differences were considered statistically significant at *P* < 0.05.

### Echocardiography

Cardiac function, chamber dilatation, and wall thickness were analyzed in 10- to 12-month-old mice by transthoracic two-dimensional (2D) and M-mode echocardiography. Measurements were carried out by a blinded operator using a high-frequency ultrasound system with a 30-MHz probe (Vevo 2100, VisualSonics Inc.). For ultrasound scans, mice were placed on a heating pad and kept under light anesthesia with isoflurane adjusted to obtain a target heart rate of 500 ± 50 bpm. Left ventricular (LV) ejection fraction and LV end-diastolic volume were obtained from the long-axis view, and LV posterior wall in diastole was obtained from the short-axis view. Right ventricular systolic function was assessed indirectly from the tricuspid annular plane systolic excursion and estimated from maximum lateral tricuspid annulus movement obtained from a 2D four-chamber apical view. Images were analyzed offline by an expert using the Vevo 2100 Workstation software. From these images, cardiac output, stroke volume, and LV mass were calculated. These measurements were normalized by the tibial length of each mice.

### Statistical analysis

Sample sizes, statistical tests, and *P* values are specified in the corresponding figure legends and subsections of Methods. Statistical analyses were performed, and graphical representations were prepared in GraphPad Prism version 8. All statistical tests were performed using two-tailed unpaired Student’s *t* tests (subjected to Welch’s correction in Fig. 4A), and numerical data are presented as means ± SD (*P < 0.05, **P < 0.01, and ***P < 0.001). No statistical method was used to predetermine sample size.

## Notes

View/request a protocol for this paper from *Bio-protocol*.
