# Supplementary material for: Adhesion G protein–coupled receptor Gpr126/Adgrg6 is essential for placental development
Source: Sci Adv. 2021 Nov 12;7(46):eabj5445. doi: 10.1126/sciadv.abj5445 (PMC8589310; doi:10.1126/sciadv.abj5445)
Supplement: Supplementary file 1 — Figs. S1 to S14 [file sciadv.abj5445_sm.pdf]

Science Advances
AAAS

Supplementary Materials for

Adhesion G protein–coupled receptor Gpr126/Adgrg6 is essential for placental development

Rebeca Torregrosa-Carrión, Rebeca Piñeiro-Sabarís, Marcos Siguero-Álvarez, Joaquím Grego-Bessa, Luis Luna-Zurita, Vitor Samuel Fernandes, Donal MacGrogan, Didier Y. R. Stainier, José Luis de la Pompa*

*Corresponding author. Email: jlpompa@cnic.es

Published 12 November 2021, *Sci. Adv.* **7**, eabj5445 (2021)
DOI: 10.1126/sciadv.abj5445

The PDF file includes:

Figs. S1 to S14

Other Supplementary Material for this manuscript includes the following:

Tables S1 to S5
Source data

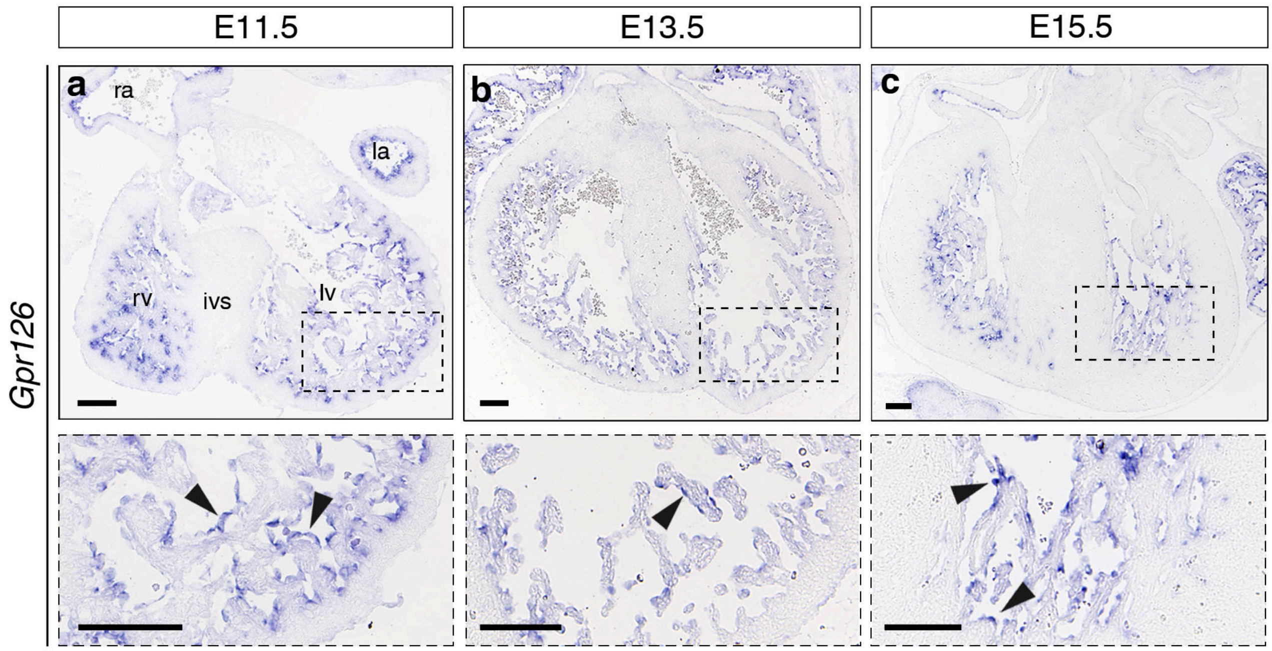

Torregrosa et al_**Supplementary Figure 1**

Supplementary Figure 1. *Gpr126* is expressed in the chamber endocardium during heart development. (a-c) ISH of *Gpr126* in transverse heart sections from WT embryos at E11.5 **(a)**, E13.5 **(b),** and E15.5 **(c)**. Arrowheads indicate expression confined to the endocardium. Scale bars, 100 µm. ivs, interventricular septum; la, left atrium; lv, left ventricle; ra, right atrium; rv, right ventricle.

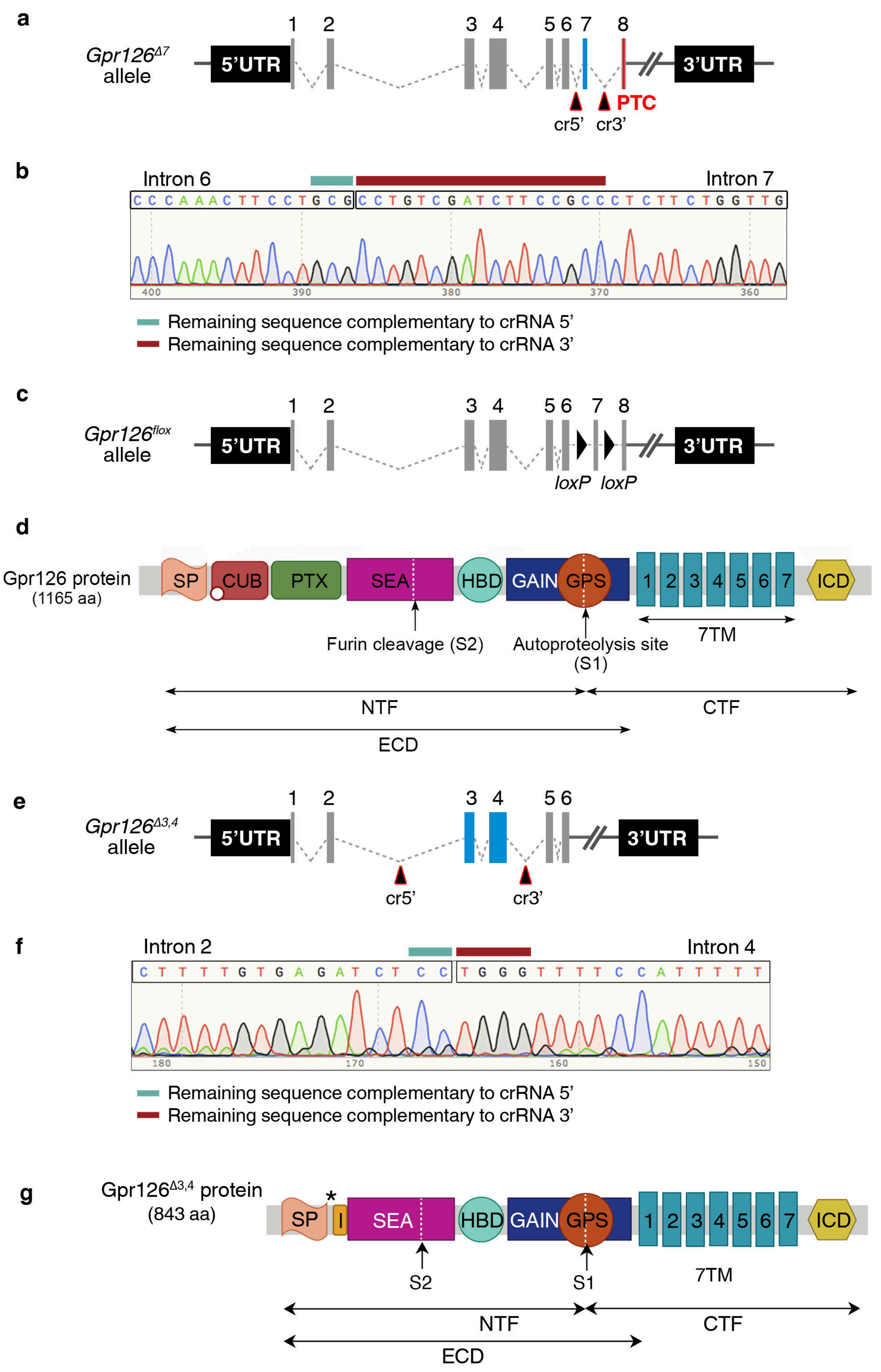

Torregrosa et al_**Supplementary Figure 2**

Supplementary Figure 2. Generation of mouse *Gpr126* mutant lines using CRISPR-Cas9. (a) Scheme of the mouse *Gpr126* locus and the 2 crRNA (5' and 3') target sites in introns 6 and 7, designed to generate the *Gpr126*$^{\Delta 7}$ mutant allele**.** The deletion of exon 7 (shown in blue) leads to a PTC in exon 8 (red). Grey boxes depict exons, dashed grey lines introns. (**b**) Chromatogram of sequencing data from F1 heterozygous *Gpr126*$^{\Delta 7/+}$ mice, showing deletion of exon 7. (**c**) Scheme of the conditional *Gpr126*^{flox} mutant allele, in which exon 7 is flanked by two loxP sites. (**d**) Gpr126 protein structure: The S2 (furin) and S1 (autoproteolysis) cleavage sites are indicated by white dashed lines. The white circle in the CUB (complement C1r/C1s, Uegf, and Bmp1-epidermal growth factor) domain depicts a calcium-binding site. SP, signal peptide; PTX, pentraxin domain; SEA, sperm protein, enterokinase and agrin domain; HBD, hormone-binding domain; GAIN, G protein-coupled receptor autoproteolysis-inducing domain; GPS, G protein-coupled receptor proteolytic site; 7TM, type II seven-transmembrane domain; ECD, extracellular domain; ICD, intracellular domain; NTF, N-terminal fragment; CTF, C-terminal fragment. **(e)** Diagram showing the 2 crRNA (5' and 3') target sites in introns 2 and 4 of the mouse *Gpr126* locus used to generate the *Gpr126*$^{\Delta 3,4}$ mutant allele**.** Targeted exons 3 and 4 are shown in blue. (**f**) Chromatogram of sequencing data from F1 heterozygous *Gpr126*$^{\Delta 3,4/+}$ mice, showing deletion of exons 3 and 4. (**g**) Structure of the hypothetical truncated Gpr126$^{\Delta 3,4}$ protein. The asterisk marks the absence of the CUB and PTX domains. Abbreviations as in (**d**).

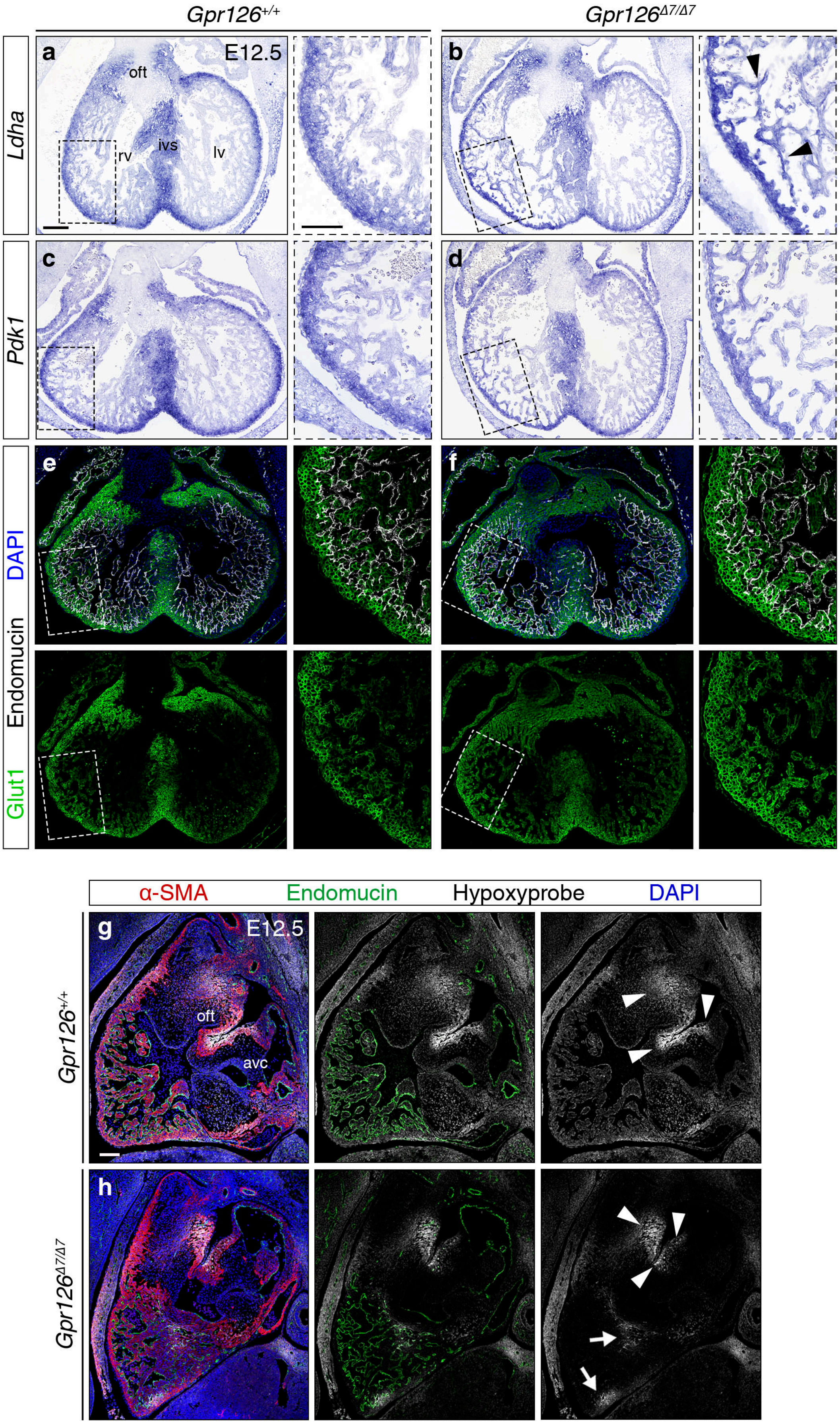

Torregrosa et al_**Supplementary Figure 3**

Supplementary Figure 3. Cardiac metabolism and hypoxic levels are unaffected in *Gpr126*$^{\Delta 7/\Delta 7}$ mutant embryonic hearts. (a-d) ISH analysis of the glycolytic enzymes *Ldha* **(a,b)** and *Pdk1* **(c,d)** in heart sections of E12.5 *Gpr126*$^{+/+}$ and *Gpr126*$^{\Delta 7/\Delta 7}$ embryos. Arrowheads in (b) indicate *Ldha* expansion to trabeculae in the right ventricle of the mutant embryo. **(e,f)** Immunostaining for GLUT1 (green), the endocardium endomucin marker (white), and the nuclear counterstain DAPI (blue) in E12.5 *Gpr126*$^{+/+}$ and *Gpr126*$^{\Delta 7/\Delta 7}$ heart sections. Magnified views of boxed areas are shown on the right. **(g,h)** Hypoxyprobe labelling of E12.5 *Gpr126*$^{+/+}$ and *Gpr126*$^{\Delta 7/\Delta 7}$ hearts. Sagittal heart sections were stained for hypoxyprobe (white, revealing hypoxic regions), alpha-smooth muscle actin to counterstain the myocardium (α-SMA) (red), endomucin (green), and DAPI (blue). Arrowheads highlight the hypoxyprobe signal in the outflow tract myocardium; arrows indicate the hypoxyprobe signal in the interventricular septum. Scale bars, 100 µm . avc, atrioventricular canal; ivs, interventricular septum; lv, left ventricle; oft, outflow tract; rv, right ventricle.

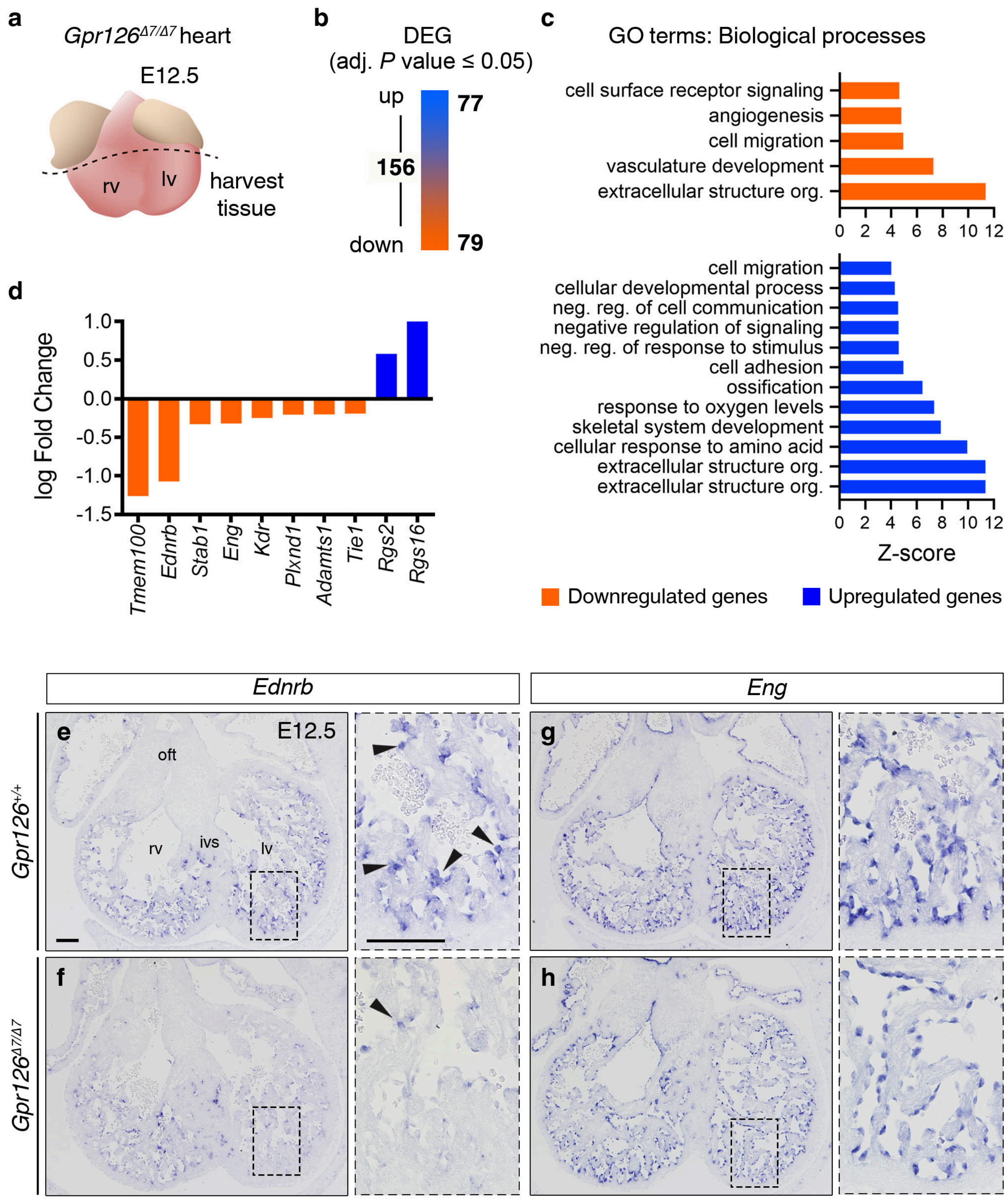

Torregrosa et al_**Supplementary Figure 4**

Supplementary Figure 4. Disruption of cardiac Gpr126 signalling affects processes related to vasculature development. (a) Cartoon illustrating the dissection of E12.5 heart ventricles (dotted line). **(b)** Chart showing the total number of differentially expressed genes (DEGs) detected by RNA-seq analysis in *Gpr126*$^{\Delta7/\Delta7}$ ventricles ($P < 0.05$). Blue indicates upregulated genes, orange downregulated genes. **(c)** GO enrichment analysis of downregulated genes (orange) and upregulated genes (blue). **(d)** Graph of log-fold change (logFC), showing the downregulation of endothelial and endocardial genes (orange) and the upregulation of regulators of G-protein signalling genes (*Rgs*). **(e-h)**. ISH analysis of *EdnrB* **(e,f)** and *Eng* **(g,h)** in E12.5 *Gpr126*$^{+/+}$ and *Gpr126*$^{\Delta7/\Delta7}$ hearts. Arrowheads indicate gene expression in the endocardium. Boxed areas are magnified on the right. Scale bars, 100 µm. ivs, interventricular septum; lv, left ventricle; oft, outflow tract; rv, right ventricle.

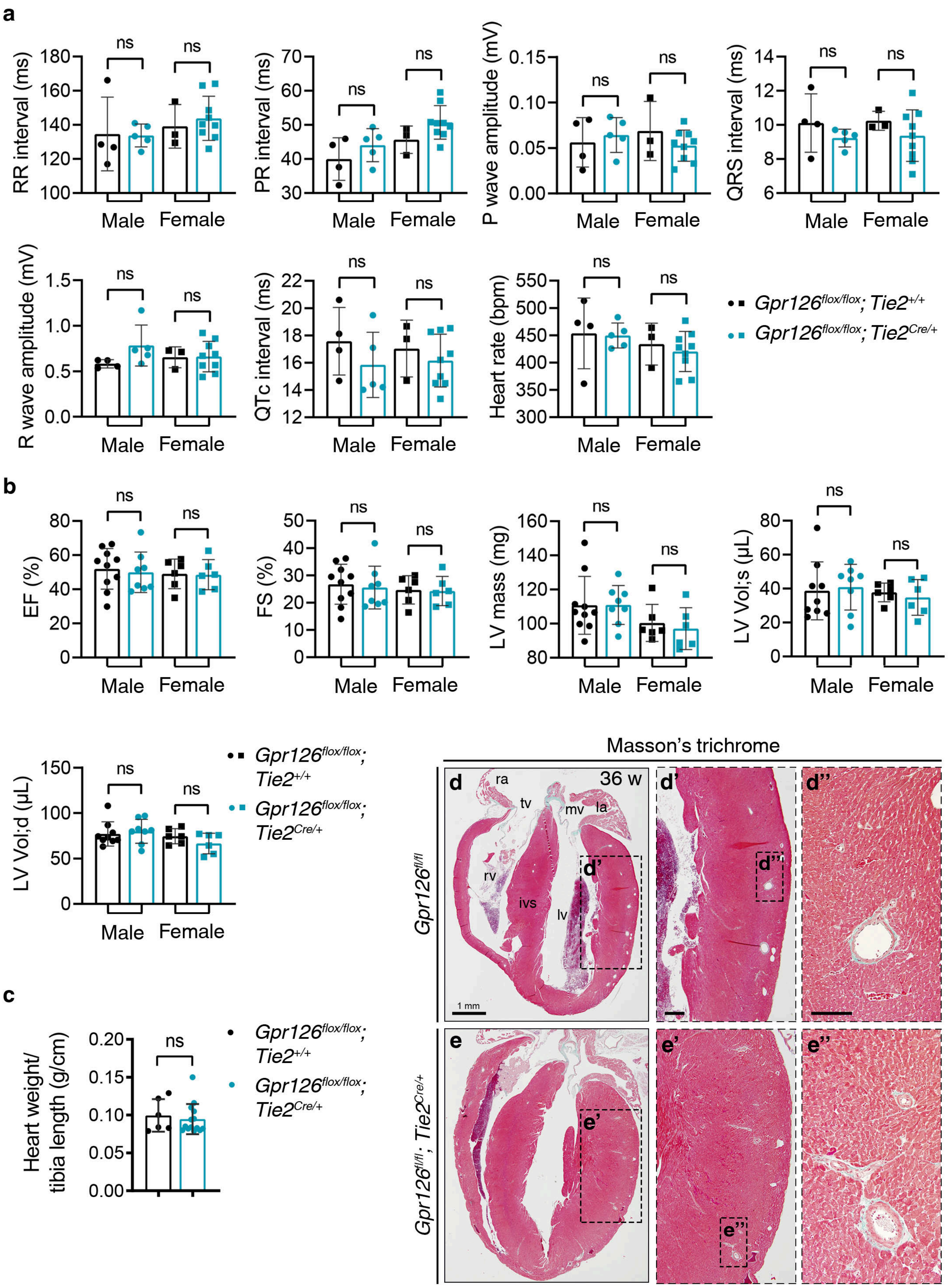

Torregrosa et al_**Supplementary Figure 5**

Supplementary Figure 5. ***Gpr126*** $^{\mathit{fl/fl}}$***;Tie2*** $^{\mathit{Cre/+}}$ **adult mice have normal cardiac morphology and cardiac function. (a)** ECG analysis of 31–36-week-old male and female *Gpr126* $^{fl/fl}$*;Tie2* $^{+/+}$ (control) and *Gpr126* $^{fl/fl}$*;Tie2* $^{Cre/+}$ adult mice. The graphs show PR interval, RR interval, P wave amplitude, QRS interval, R wave amplitude, corrected QT interval (QTc), and heart rate, with males (squares) and females (circles) considered separately. Data are means ± SD (n= 4 control and 5 mutant males; 5 control and 9 mutant females). Statistical significance was determined by unpaired Student's *t*-test with Welch's correction (ns, not significant). **(b)** Echocardiography analysis of left ventricular (LV) function in *Gpr126* $^{fl/fl}$*; Tie2* $^{+/+}$ (control) and *Gpr126* $^{fl/fl}$*; Tie2* $^{Cre/+}$ adult mice at 32-43 weeks old. The graphs show left ventricular (LV) ejection fraction (EF), fractional shortening (FS), LV mass, LV end-systolic volume (LV Vol;s) and LV end-diastolic volume (LV Vol;d), considering males (squares) and females (circles) separately. Data are presented as mean ± SD (n= 10 control and 8 mutant males; 6 control and 6 mutant females). Statistical significance was determined by unpaired Student's *t*-test (ns, not significant). **(c)** Chart depicting heart weight (mg) to tibia length (cm) ratios in 46–55-week-old *Gpr126* $^{fl/fl}$*;Tie2* $^{+/+}$ (control) and *Gpr126* $^{fl/fl}$*;Tie2* $^{Cre/+}$ adult mice. Data are means ± SD (n= 6 control and 14 mutants). Statistical significance was determined by unpaired Student's *t*-test (ns, not significant). **(d-e'')** Masson's trichrome staining of transverse heart sections from 46-week-old *Gpr126* $^{fl/fl}$ **(d-d'')** and *Gpr126* $^{fl/fl}$*;Tie2* $^{Cre/+}$ mutants **(e-e'')**. Boxed regions in **(d,e)** are magnified in **(')**; boxed regions in **(d', e')** are magnified in **('').** Scale bars, 100 µm unless otherwise indicated. ivs, interventricular septum; la, left atrium; lv, left ventricle; mv, mitral valve; ra, right atrium; rv, right ventricle; tv, tricuspid valve.

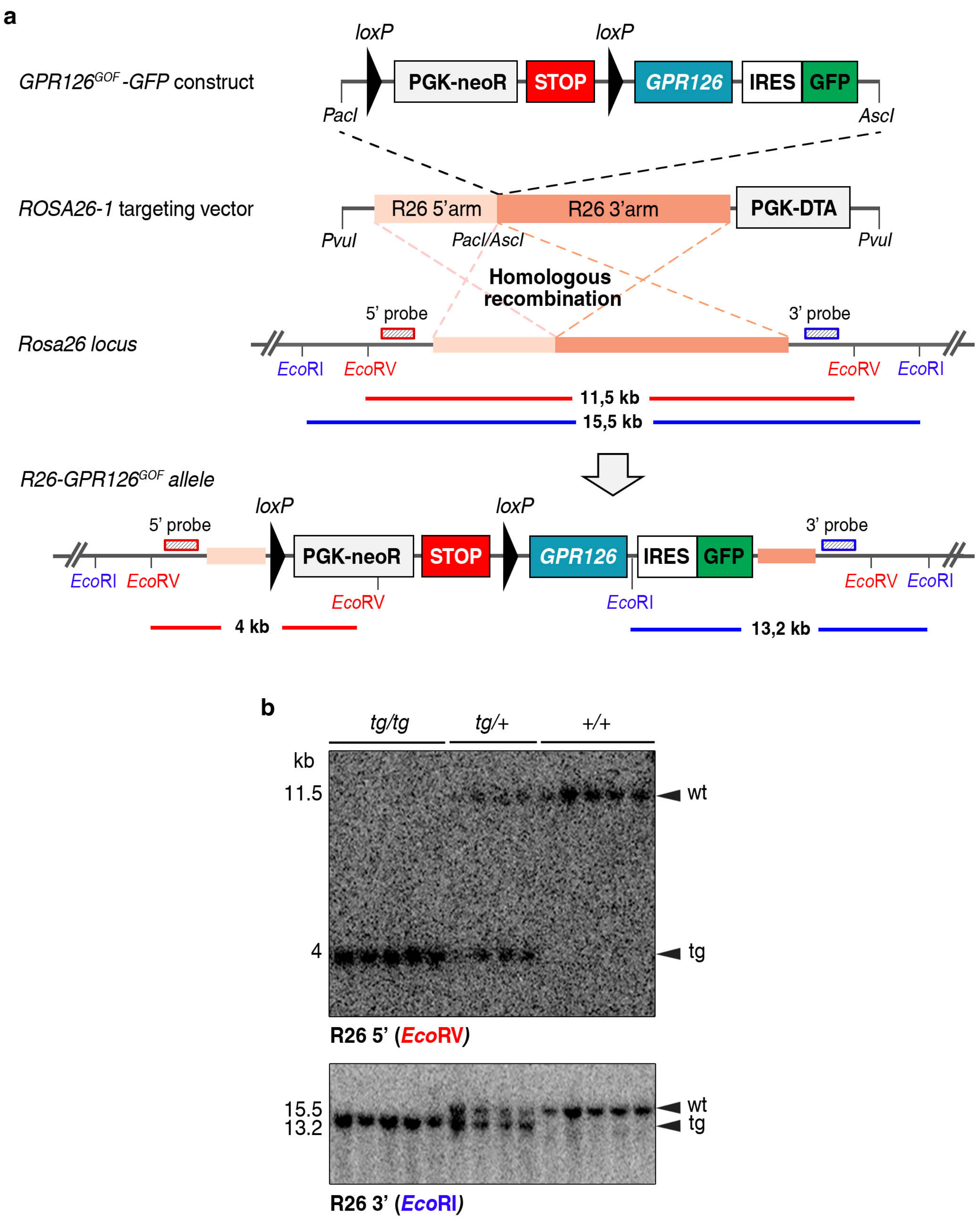

Torregrosa et al_**Supplementary Figure 6**

Supplementary Figure 6. Gene targeting of the *Rosa2*6 locus to generate the conditional *R26-GPR126^{GOF}-GFP* transgenic line. (a) Gene targeting strategy. From top to bottom: modified *pROSA26-1* plasmid vector harbouring the *loxP-PGK-NeoR-STOP-loxP-GPR126-IRES-eGFP* construct between the *Rosa26* 5' and 3' homology arms. Targeted *Rosa26* locus, showing the location of the 5' and 3' hybridization probes used for Southern analysis and the *Ec*o*R*V and *EcoR*I sites. The *R26-GPR126^{GOF}* allele generated by homologous recombination in ES cells. PGK, phosphoglycerate kinase; neoR, neomycin resistance gene; IRES, internal ribosome entry site; GFP, green fluorescent protein; DTA, diphtheria toxin. **(b)** Southern blot analysis of *EcoR*V-digested DNA (top panel) and *EcoR*I-digested DNA (bottom panel) from homozygous (*tg/tg*), heterozygous (*tg*/+), and WT (+/+) *GPR126^{GOF}* offspring.

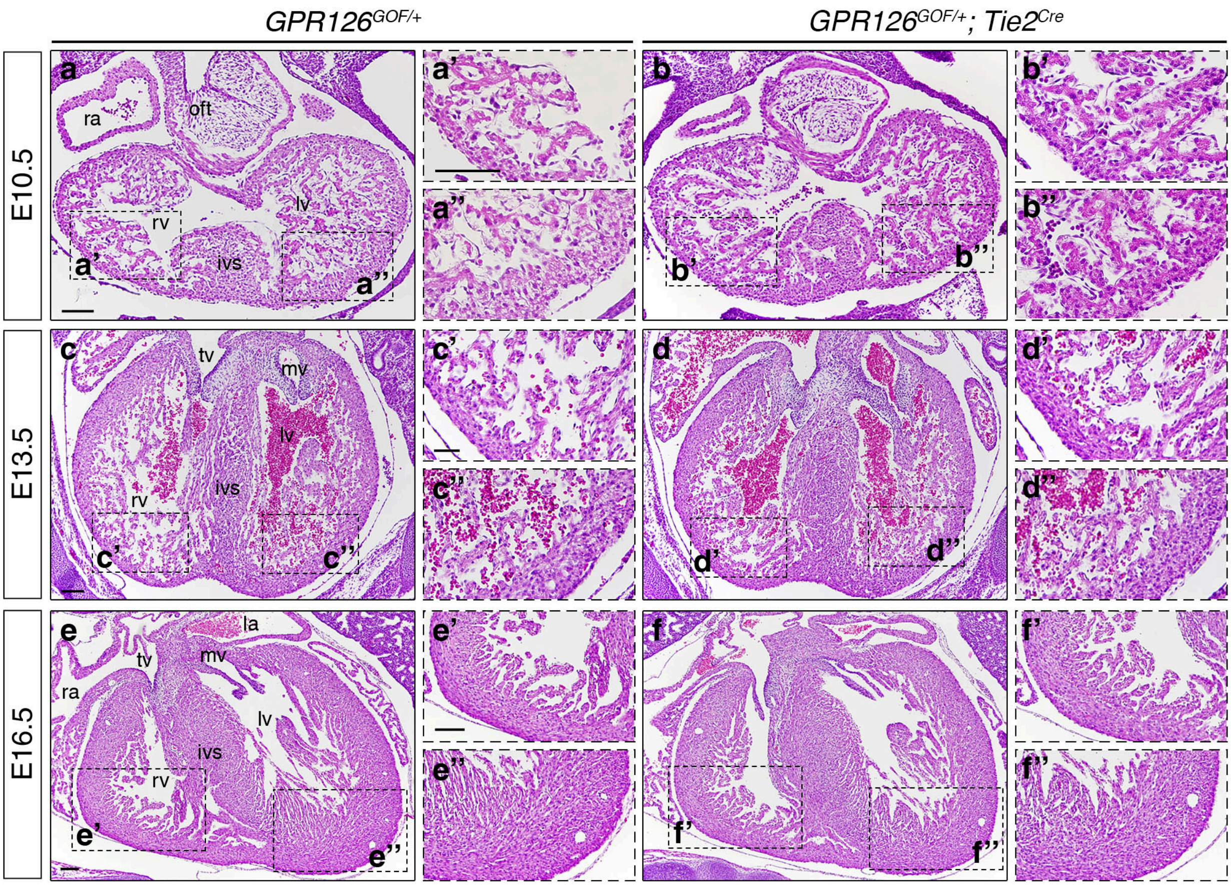

Torregrosa et al_**Supplementary Figure 7**

Supplementary Figure 7. Conditional *GPR126* overexpression in the *Tie2-Cre* lineage allows normal heart development. (a-f'') H&E staining of transverse heart sections from $GPR126^{GOF/+}$ (control) and $GPR126^{GOF/+};Tie2^{Cre/+}$ embryos at E10.5 **(a-b'')**, E13.5 **(c-d'')**, and E16.5 **(e-f'')**. Magnified views of the right ventricle are shown in **(')**, and of the left ventricle in **('')**. Scale bars, 100 µm. ivs, interventricular septum; la, left atrium; lv, left ventricle; mv, mitral valve; oft, outflow tract; ra, right atrium; rv, right ventricle; tv, tricuspid valve.

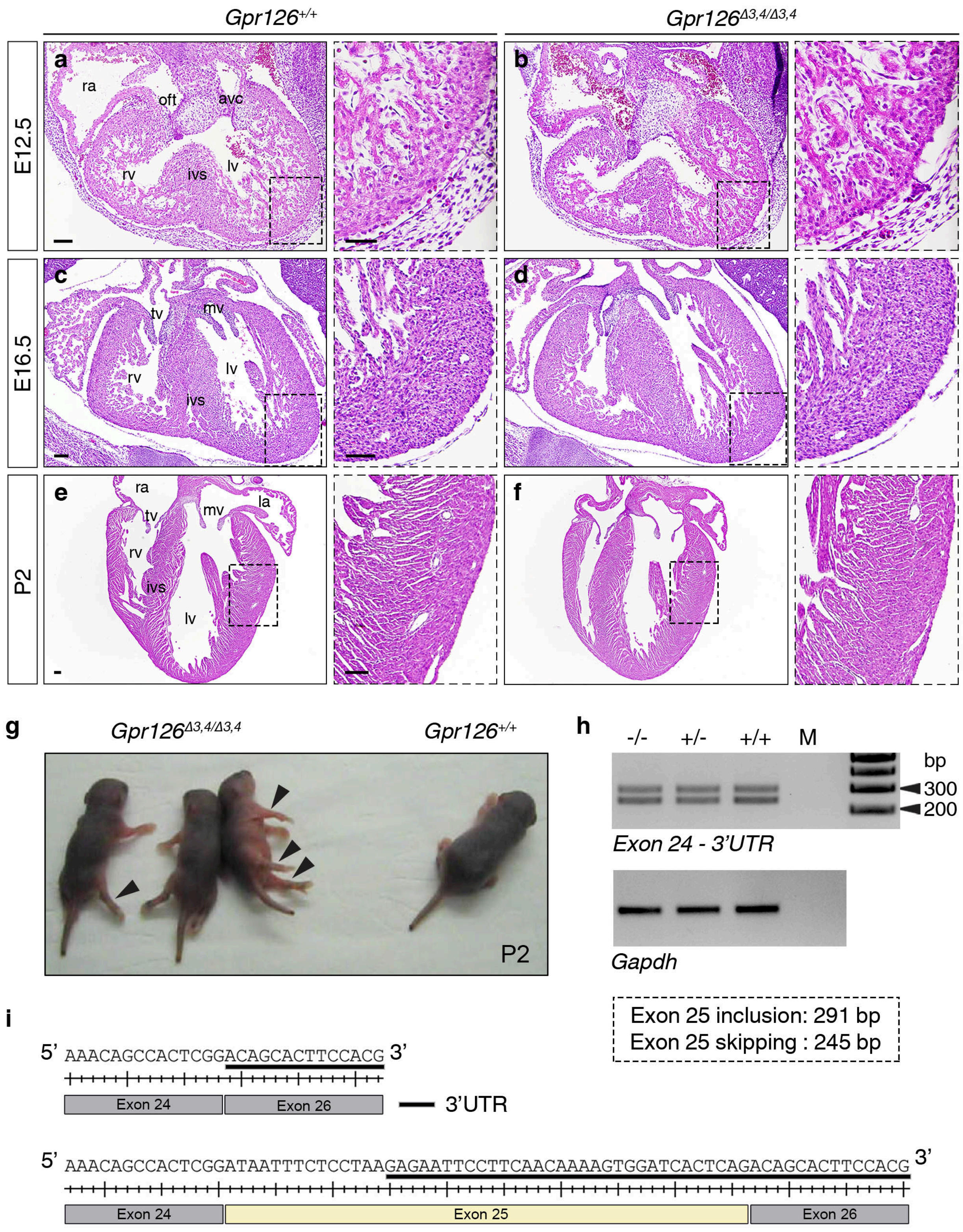

Torregrosa et al_**Supplementary Figure 8**

Supplementary Figure 8. *Gpr126*$^{\Delta 3,4}$ mutants show defects in PNS development, but not in the heart. (a-f) H&E staining of *Gpr126*$^{+/+}$ and *Gpr126*$^{\Delta 3,4/\Delta 3,4}$ heart sections at E12.5 **(a,b)**, E16.5 **(c,d)**, and P2 **(e,f)**. Boxed regions are shown at high magnification to the right of each panel. Scale bars, 100 µm. avc, atrioventricular canal; ivs, interventricular septum; lv, left ventricle; oft, outflow tract; ra, right atrium; rv, right ventricle. **(g)** *Gpr126*$^{+/+}$ and *Gpr126*$^{\Delta 3,4/\Delta 3,4}$ littermates at P2. Arrowheads point to joint contractures and rigid limbs in the mutants. **(h)** RT-PCR of *Gpr126* in *Gpr126*$^{+/+}$, *Gpr126*$^{\Delta 3,4/+}$ and *Gpr126*$^{\Delta 3,4/\Delta 3,4}$ embryos at E9.5. Primers span from exon 24 to the 3'UTR region. The two RT-PCR products represent two *Gpr126* transcript isoforms based on the inclusion or exclusion of exon 25 as an alternative spliced exon. Relative expression was normalized to the housekeeping gene *Gapdh*. **(i)** Sequencing analysis of the two transcript variants shown in **(h)**.

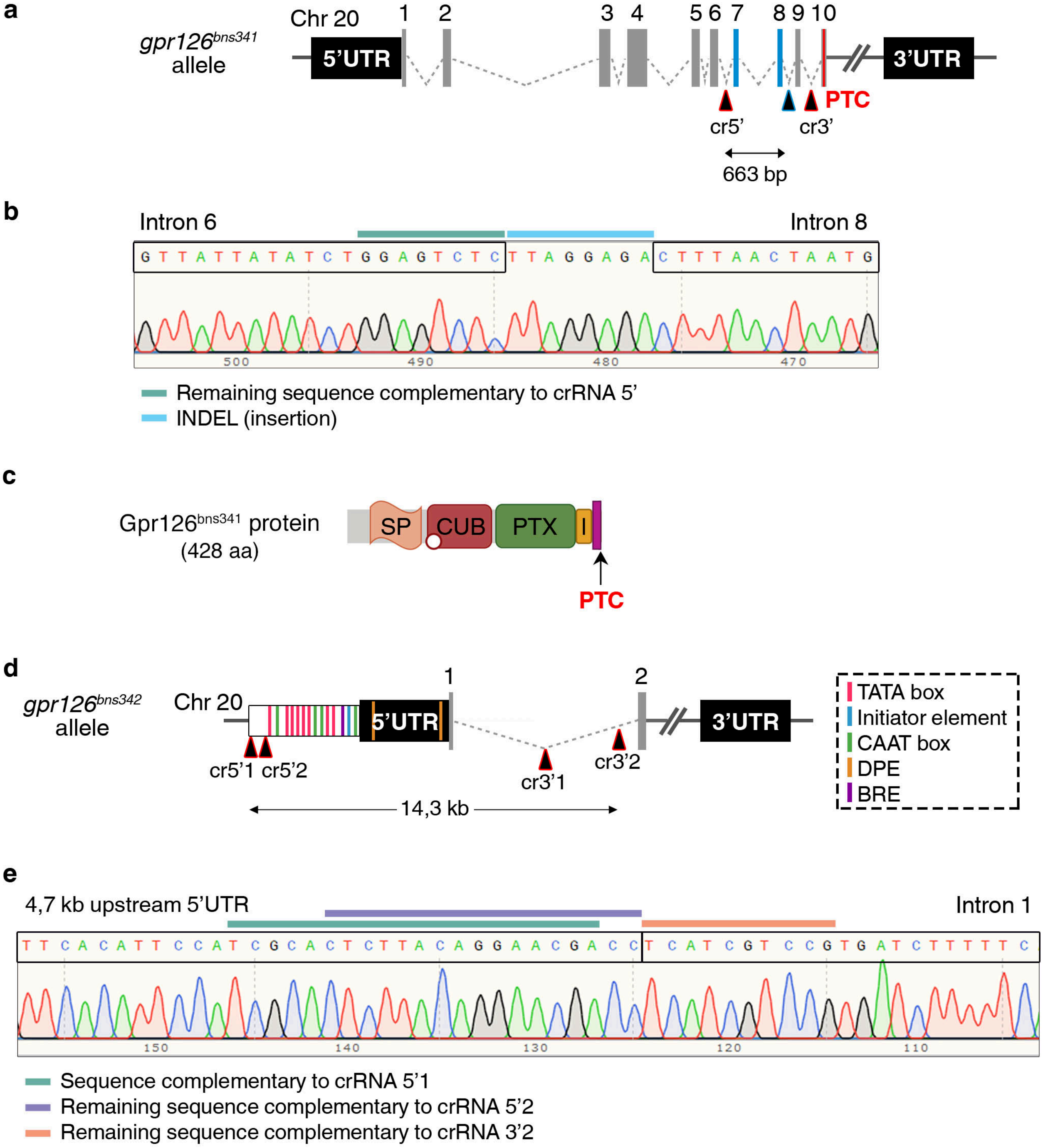

Torregrosa et al_**Supplementary Figure 9**

Supplementary Figure 9. Generation of zebrafish *gpr126* mutant lines using CRISPR-Cas9. (a) Diagram showing 2 crRNA (5' and 3') target sites in introns 6 and 9 of the zebrafish *gpr126* locus, which resulted in deletion of exons 7 and 8 (shown in blue) to generate the $gpr126^{bns341}$ mutant allele**.** Deletion of exons 7-8 leads to a PTC in exon 10 (shown in red). Grey boxes depict exons, dashed grey lines illustrate introns. **(b)** Chromatogram of sequencing data from F1 heterozygous $gpr126^{bns341/+}$ fish, showing deletion of exons 7 and 8, plus an 8 bp insertion (indel). **(c)** Scheme of the predicted $Gpr126^{bns341}$ protein, formed by 428 aa. PTC, premature termination codon. Functional domains are as described in *Suppl. Fig. 2d*. **(d)** Diagram showing the 4 crRNA (5'1, 5'2, 3'1, 3'2) target sites in the promoter region of the zebrafish *gpr126* locus used to generate the $gpr126^{bns342}$ mutant allele**.** Grey boxes depict exons, dashed grey lines illustrate introns. DPE, downstream promoter element; BRE, B recognition element. **(e)** Chromatogram of sequencing data from F1 heterozygous $gpr126^{bns342/+}$ fish, showing deletion of the targeted promoter region.

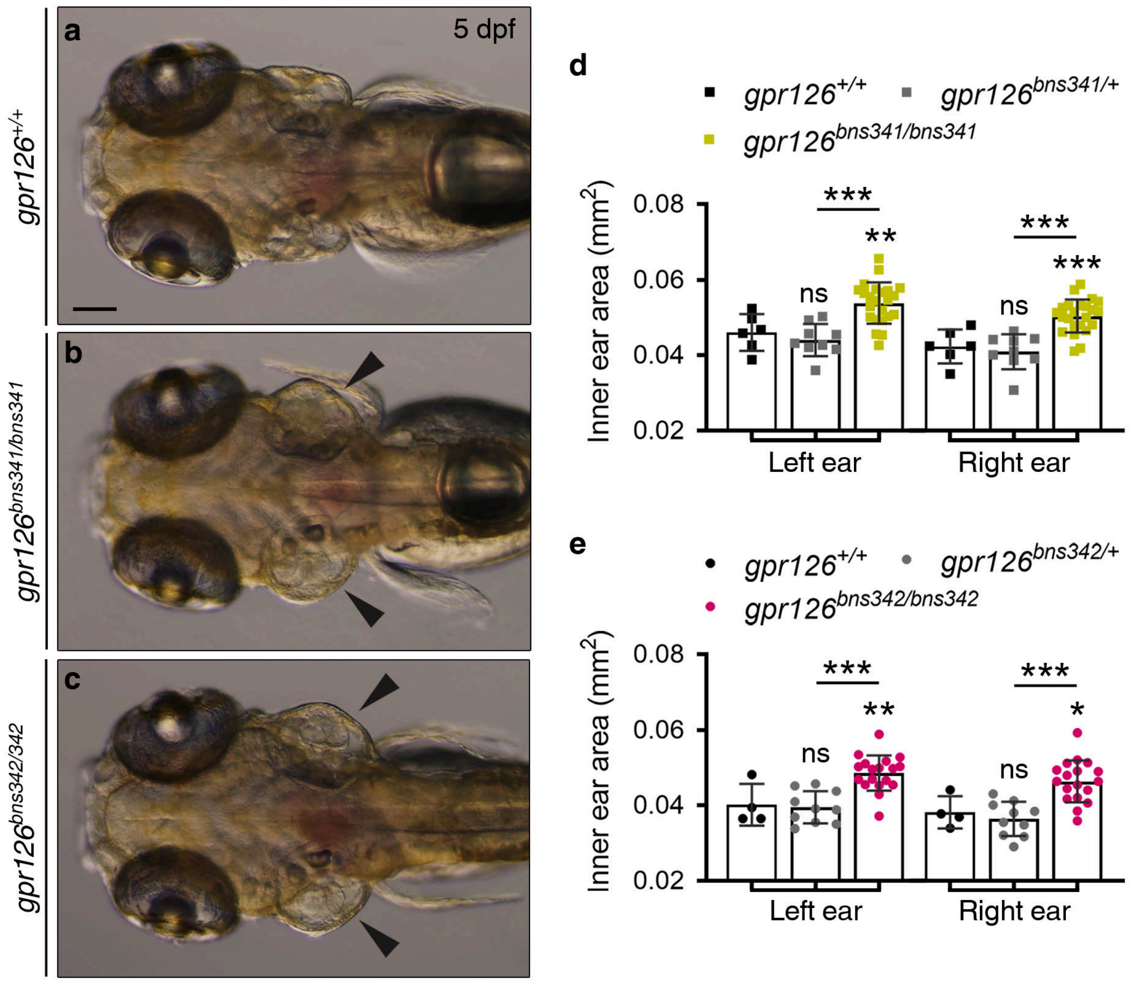

Torregrosa et al_**Supplementary Figure 10**

Supplementary Figure 10. The *gpr126*^{bns341} and *gpr126*^{bns342} mutations disrupt inner ear development in zebrafish. (a-c) Dorsal view of *gpr126*$^{+/+}$ **(a)**, *gpr126*$^{bns341/bns341}$ **(b)**, and *gpr126*$^{bns342/bns342}$ **(c)** zebrafish at 5 dpf. Arrowheads mark swollen ears in the mutants. Scale bar, 100 µm. **(d,e)** Quantification of left and right ear area in WT, heterozygous and homozygous *gpr126*^{bns341} **(d)** and *gpr126*^{bns342} **(e)** zebrafish mutants. Data are means ± SD. Statistical significance was determined by unpaired Student's *t*-test (ns, not significant, *$P < 0.05$; **$P < 0.01$, ***$P < 0.001$).

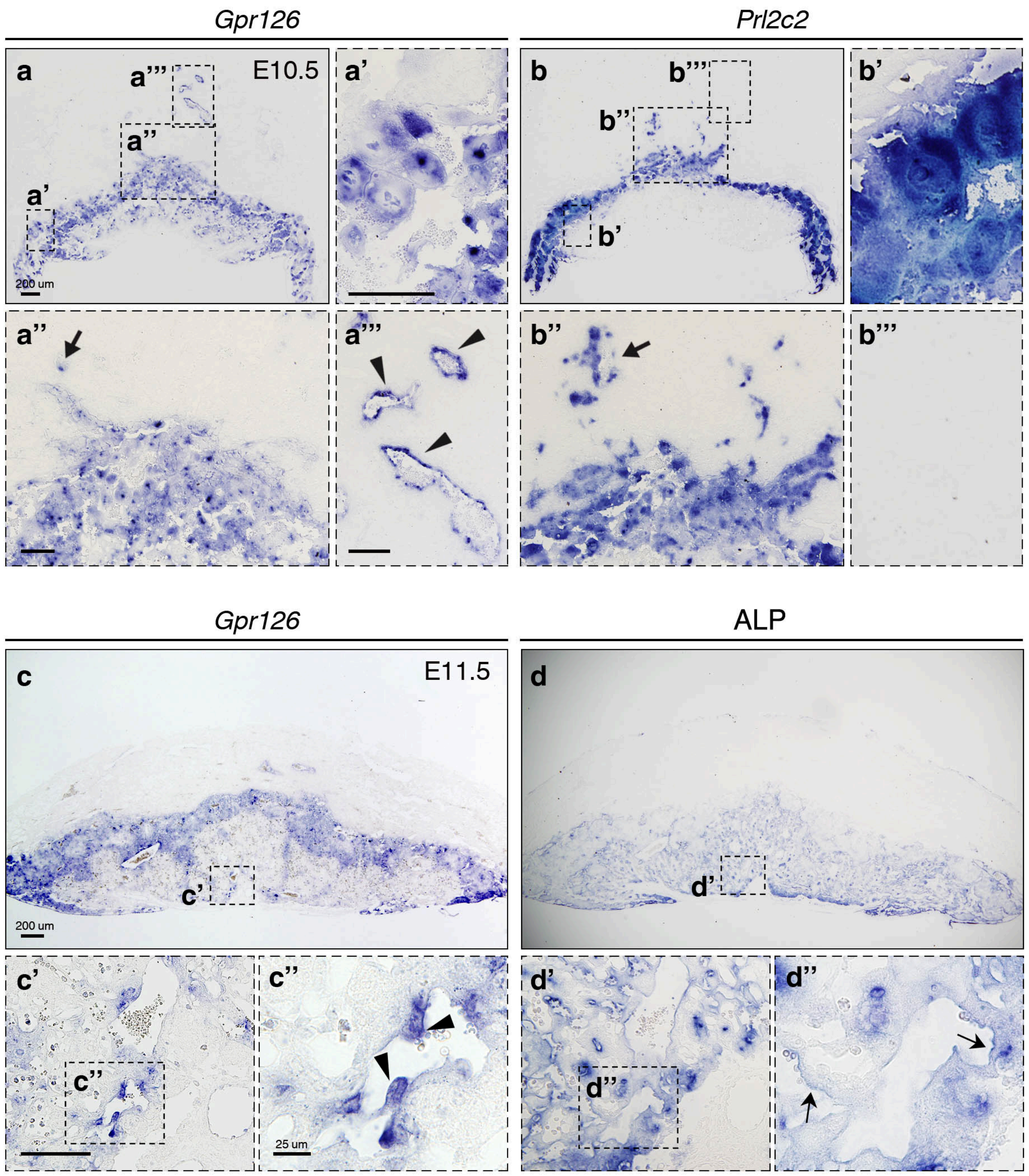

Torregrosa et al. **Supplementary Figure 11**

Supplementary Figure 11. Comparison of *Gpr126* expression with placental trophoblast markers by ISH analysis. (a-b''') ISH of *Gpr126* **(a-a''')** and *Prl2c2/Plf* **(b-b''')** on sagittal sections of E11.5 WT placentas. High power magnification views corresponding to boxed areas show P-TGCs **(')**, the border between the decidua and the junctional zone **('')**, and the maternal spiral arteries **(''')**. Arrowheads in **(a''')** indicate *Gpr126*-positive maternal endothelium, which is negative for *Prl2c2* expression **(b''')**. **(c-d'')** ISH of *Gpr126* **(c-c'')** and staining of alkaline phosphatase activity (ALP) **(d-d'')** on sagittal sections of E11.5 WT placentas. Boxed regions in **(c,d)** are magnified in **(')**; boxed regions in **(c',d')** are magnified in **('')**. Arrowheads in **(c'')** point to *Gpr126* expression in trophoblasts protruding into maternal blood sinuses, likely S-TGC. Arrows in **(d'')** indicate the endogenous alkaline phosphatase activity of the syncytiotrophoblast layer underneath. Scale bars, 100 μm; otherwise it is indicated.

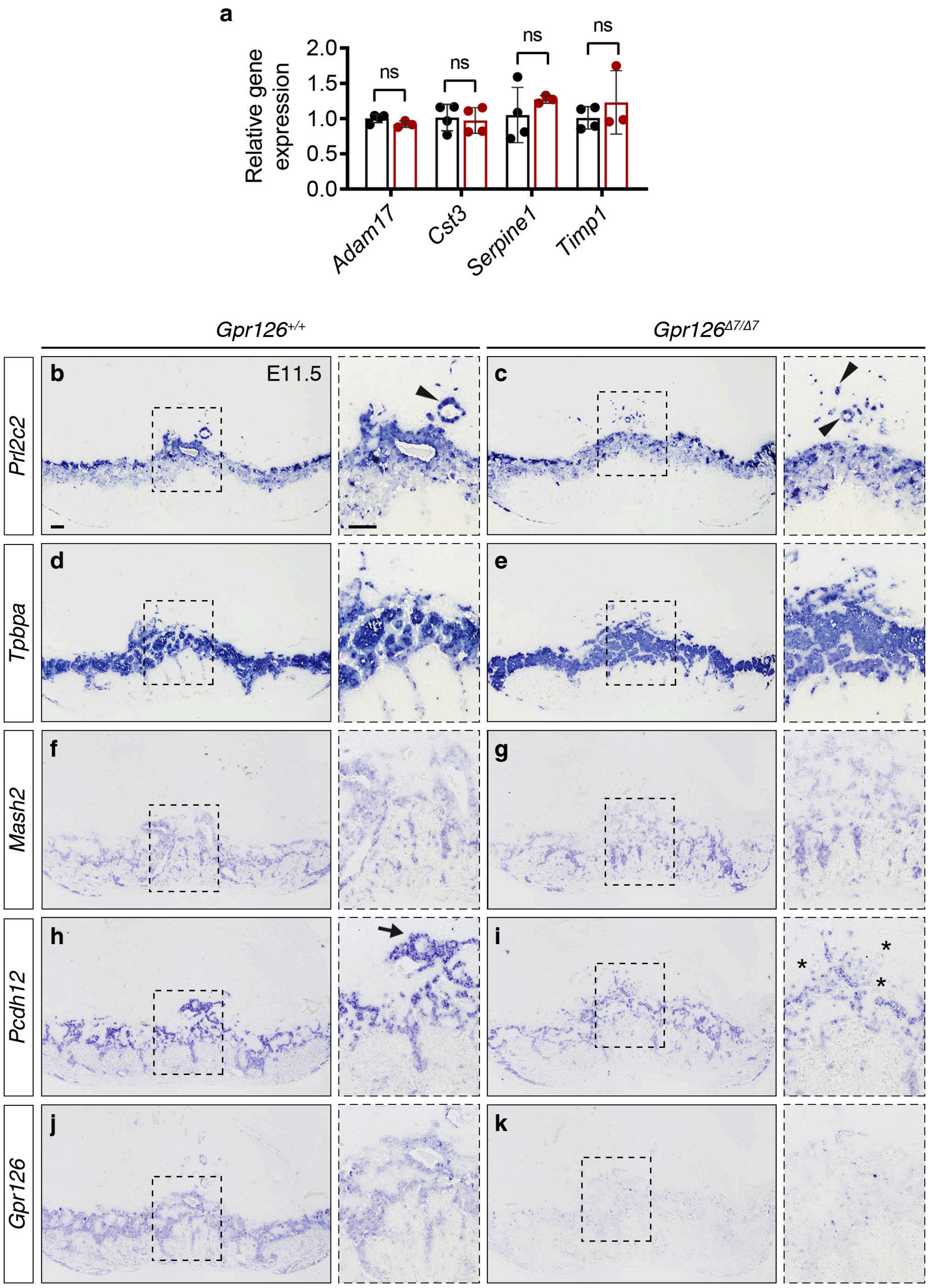

Torregrosa et al_**Supplementary Figure 12**

Supplementary Figure 12. ISH gene expression analysis of trophoblast-subtype associated markers and preeclampsia-associated factors in *Gpr126*$^{\Delta7/\Delta7}$ placentas. (a) qRT-PCR showing relative gene expression of known preeclampsia-associated proteases and protease inhibitors. *B-actin* was used as a housekeeping gene. Data are means ± SD (n= 4 WT and 3 or 4 mutant placentas). Statistical significance was determined by unpaired Student's *t*-test (ns, not significant). **(b-k)** ISH of *Prl2c2/Plf* **(b-c)**, *Tpbpa* **(d-e)**, *Mash2/Ascl2* **(f-g)**, *Pcdh12* **(h-i),** and *Gpr12*6 **(j-k)** on sagittal sections of E11.5 *Gpr126*$^{+/+}$ and *Gpr126*$^{\Delta7/\Delta7}$ placentas. High power magnification views corresponding to boxed areas are shown on the right. Arrowheads in **(b,c)** indicate *Prl2c2*-positive Spa-TGCs; arrows in **(h)** mark multi-layered expression of *Pcdh-12*-positive Spa-TGCs; asterisks in **(i)** indicate patchy expression of *Pcdh12*. Scale bars, 200 µm.

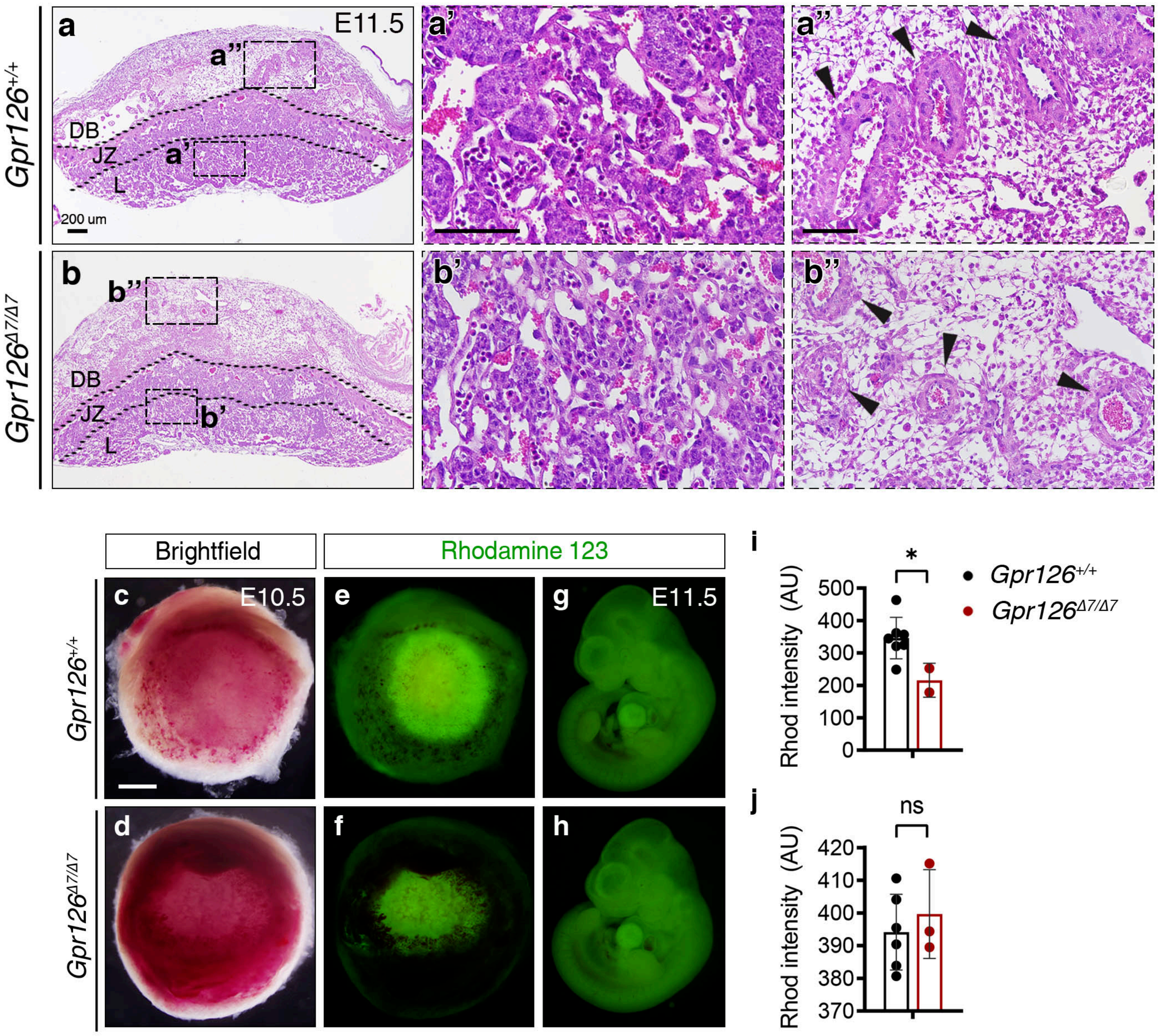

Torregrosa et al_**Supplementary Figure 13**

Supplementary Figure 13. ***Gpr126***$^{\Delta7/\Delta7}$ **mutant placentas show blood accumulation in the labyrinth. (a-b'')** H&E staining on E11.5 *Gpr126*$^{+/+}$ **(a)** and *Gpr126*$^{\Delta7/\Delta7}$ **(b)** sagittal placental sections. The black dotted lines delineate the different placental layers. The panels to the right **(',")** are high magnification views of the boxed areas, showing the labyrinth **(')** and the maternal decidua **(**with maternal spiral arteries marked with arrowheads). **(c,d)** Whole-mount images of E10.5 *Gpr126*$^{+/+}$ **(c)** and *Gpr126*$^{\Delta7/\Delta7}$ **(d)** mouse placentas. **(e,f**) Whole-mount images of intracellular accumulation of rhodamine 123 in the labyrinth of E10.5 *Gpr126*$^{+/+}$ **(e)** and *Gpr126*$^{\Delta7/\Delta7}$ **(f)** placentas. Scale bar, 1 mm. **(g,h)** Whole-mount images of the transplacental passage of rhodamine 123 in E11.5 *Gpr126*$^{+/+}$ **(g)** and *Gpr126*$^{\Delta7/\Delta7}$ **(h)** embryos. **(i,j)** Quantification of rhodamine 123 fluorescence intensity (AU) in E10.5 *Gpr126*$^{+/+}$ and *Gpr126*$^{\Delta7/\Delta7}$ placentas **(i)** and E11.5 *Gpr126*$^{+/+}$ and *Gpr126*$^{\Delta7/\Delta7}$ embryos **(j)**. Data are means ± SD (n= 7 *Gpr126*$^{+/+}$ and 2 *Gpr126*$^{\Delta7/\Delta7}$ placentas; n= 6 *Gpr126*$^{+/+}$ and 3 *Gpr126*$^{\Delta7/\Delta7}$ embryos). Statistical significance was determined by unpaired Student's *t*-test (ns, not significant; $^{*}P < 0.05$). Scale bars, 100 μm, otherwise it is indicated. DB, decidua basalis; JZ, junctional zone; L, labyrinth.

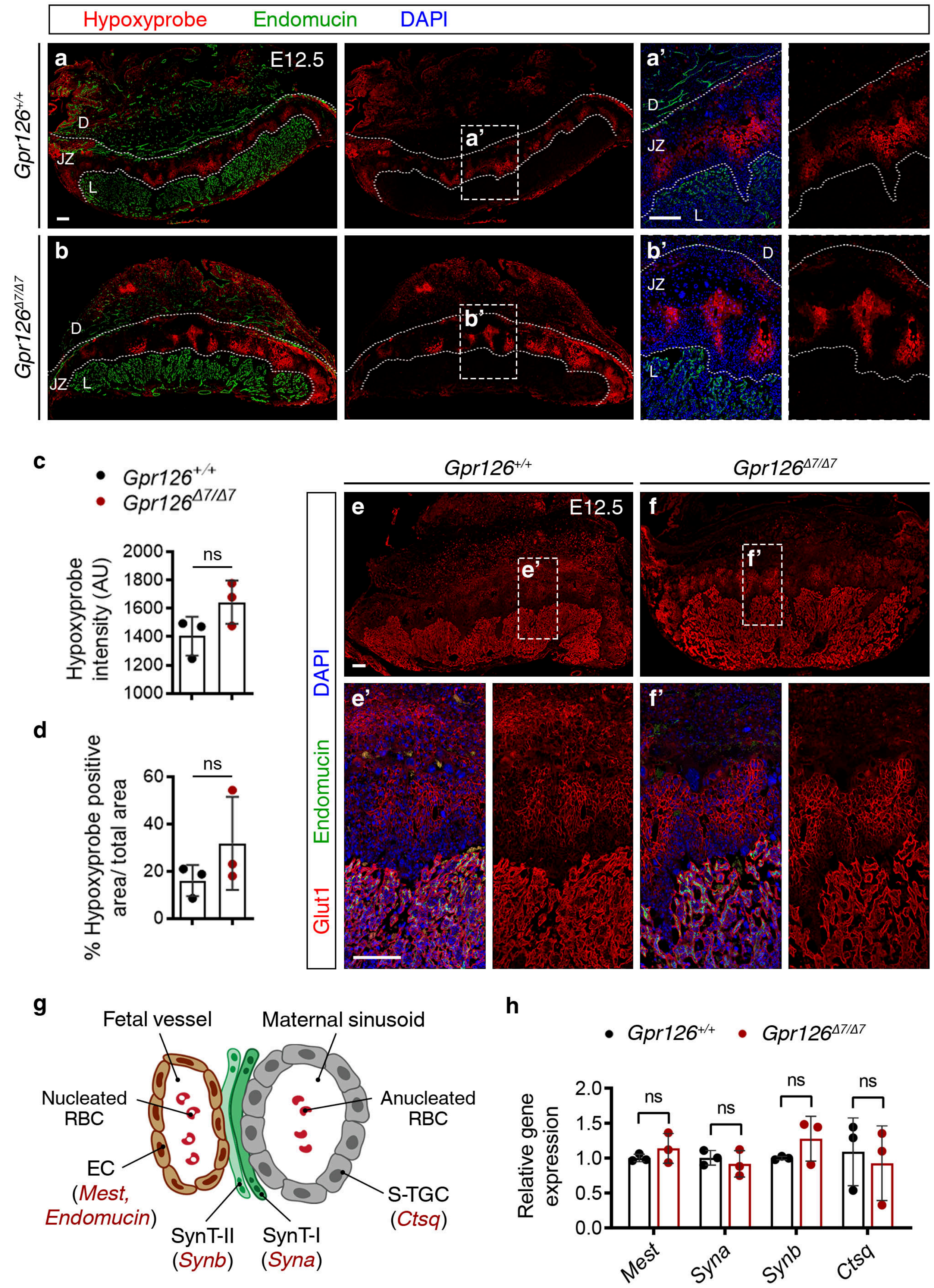

Torregrosa et al_**Supplementary Figure 14**

Supplementary Figure 14. Placental transport is unaffected in *Gpr126*$^{\Delta7/\Delta7}$ placentas. (a-b') Hypoxyprobe labelling (red) of sagittal sections from E12.5 *Gpr126*$^{+/+}$ **(a, a')** and *Gpr126*$^{\Delta7/\Delta7}$ placentas **(b, b')**. Sections were counterstained with endomucin (green) and DAPI (blue). Boxed areas are shown at high magnification on the right **(')**. The white dotted lines delineate the decidua (D), junctional zone (JZ), and labyrinth (L) layers. **(c,d)** Quantification of hypoxyprobe mean thresholded intensity levels (AU) **(c)** and the area of hypoxyprobe-positive staining as a proportion of total placental area (ratio) **(d)** in E12.5 *Gpr126*$^{+/+}$ and *Gpr126*$^{\Delta7/\Delta7}$ placentas. Data are means ± SD (n= 3 *Gpr126*$^{+/+}$ and 3 *Gpr126*$^{\Delta7/\Delta7}$ placentas). Statistical significance was determined by unpaired Student's *t*-test (ns, not significant). **(e-f')** Immunostaining for GLUT1 (red) and the endothelial marker endomucin (green) in E12.5 *Gpr126*$^{+/+}$ **(e, e')** and *Gpr126*$^{\Delta7/\Delta7}$ **(f,f')** sagittal placental sections. Nuclei were counterstained with DAPI (blue). Boxed areas are shown at high magnification beneath the main panels **(')**. Scale bars, 200 µm. **(g)** Cartoon illustrating the interhemal barrier separating foetal blood vessels and maternal sinusoids, indicating marker gene expression. EC, endothelial cells; RBC, red blood cells; SynT-II, syncytiotrophoblast II; SynT-I, syncytiotrophoblast I; S-TGC, sinusoidal trophoblast giant cells. **(h)** qRT-PCR showing relative gene expression of the foetal endothelial marker *Mest,* the syncytiotrophoblast markers *Syna* and *Synb*, and the S-TGC marker *Ctsq* in E11.5 *Gpr126*$^{+/+}$ and *Gpr126*$^{\Delta7/\Delta7}$ placentas. *B-actin* was used as a housekeeping gene. Data are means ± SD (n= 3 WT and 3 mutant placentas). Statistical significance was determined by unpaired Student's *t*-test (ns, not significant).

Supplementary Tables

Supplementary Table 1. Viability analysis of *Gpr126* genetically modified mice resulting from different intercrosses. Sheet 1: Genotype analysis of mouse embryos derived from heterozygous *Gpr126*$^{\Delta7/+}$ intercrosses. **Sheet 2:** Genotype analysis of embryos derived from crosses of *Gpr126*$^{fl/+}$;*Nfatc1*$^{panCre/+}$ males and *Gpr126*$^{fl/fl}$ females. **Sheet 3:** Genotype analysis of embryos derived from crosses of *Gpr126*$^{fl/+}$;*Tie2*$^{Cre/+}$ males and *Gpr126*$^{fl/fl}$ females. **Sheet 4:** Genotype analysis of embryos derived from crosses of *Gpr126*$^{fl/+}$;*Mesp1*$^{Cre/+}$ males and *Gpr126*$^{fl/fl}$ females. **Sheet 5:** Genotype analysis of embryos derived from crosses of *Gpr126*$^{\Delta7/+}$*;Tie2*$^{Cre/+}$ males and *Gpr126*$^{fl/fl}$ females. **Sheet 6:** Genotype analysis of embryos derived from crosses of *Gpr126*$^{\Delta7/+}$*;Mesp1*$^{Cre/+}$ males and *Gpr126*$^{fl/fl}$ females. **Sheet 7:** Genotype analysis of embryos derived from crosses of *Tie2*$^{Cre/Cre}$ males and *GPR126*$^{GOF/+}$ females. **Sheet 8:** Genotype analysis of embryos derived from crosses of *Gpr126*$^{\Delta7/+}$*;Tie2*$^{Cre/+}$ males and *GPR126*$^{GOF/+}$*;Gpr126*$^{\Delta7/+}$ females. **Sheet 9:** Genotype analysis of embryos derived from heterozygous *Gpr126*$^{\Delta3,4/+}$ intercrosses. **Sheet 10:** Genotype analysis of embryos derived from crosses of *Gpr126*$^{fl/+}$; *Sox2*$^{Cre/+}$ males and *Gpr126*$^{fl/fl}$ females. **Sheet 11:** Genotype analysis of embryos derived from crosses of *Gpr126*$^{fl/fl}$ males and *Gpr126*$^{fl/+}$;*Sox2*$^{Cre/+}$ females. **Sheet 12:** Genotype analysis of embryos derived from crosses of *Gpr126*$^{\Delta7/+}$;*Sox2*$^{Cre/+}$ males and *GPR126*$^{GOF/GOF}$;*Gpr126*$^{\Delta7/+}$ females. **Sheet 13:** Genotype analysis of embryos derived from crosses of *GPR126*$^{GOF/GOF}$; *Gpr126*$^{\Delta7/+}$ males and *Gpr126*$^{\Delta7/+}$;*Sox2*$^{Cre/+}$ females.

Supplementary Table 2. RNA-seq analysis of *Gpr126*$^{\Delta7/\Delta7}$ versus *Gpr126*$^{+/+}$ ventricles at E12.5. List of the differentially expressed genes (DEG) identified by RNA-seq ($P < 0.05$). Upregulation is shown in orange and downregulation in blue.

Supplementary Table 3. GO enrichment analysis of DEG genes. Overrepresented GO terms and KEGG pathways for the differentially upregulated (red) and downregulated genes (blue). GO terms are listed by the Z-score value. Data are the standard output from GO Elite.

Supplementary Table 4. Sequence comparisons of *Gpr126* zebrafish and mouse genes. Sheet 1: Sequence similarity analysis of zebrafish *gpr126*. **Sheet 2:** Sequence similarity analysis of mouse *Gpr126*.

Supplementary Table 5. CRISPR-Cas9 reagents, genotyping and probes primers, and antibodies. Sheet1: CRISPR-Cas9 elements used for the generation of mouse and zebrafish lines. Top: Sequences of Alt-R tracrRNA, crRNAs, and ssODNs used to generate *Gpr126*$^{\Delta3,4}$, *Gpr126*$^{\Delta7}$, and *Gpr126*^{flox} mouse lines by CRISPR/Cas9 technology. Bottom: Sequences of

constant and crRNA oligos used to generate *gpr126*^{bns341} and *gpr126*^{bns342} zebrafish mutants by CRISPR/Cas9 technology. crRNA, crispr RNA; tracrRNA, trans-activating crispr RNA; ssODN, single-stranded oligodeoxynucleotide. **Sheet 2:** List of primers used for genotyping of mouse lines (top) and zebrafish lines (bottom). Ann, annealing; E, exon; I, intron; T^{a}, temperature; For, forward; Rev, reverse; mut, mutated; tg, transgene. **Sheet 3:** List of primers used for mRNA probe generation. **Sheet 4:** List of primary and secondary antibodies used in this study. **Sheet 5:** List of qRT-PCR primers used in this study.
